# The Resettlement Journey: Understanding The Role of Social Connectedness on Well-being and Life Satisfaction among (Im)migrants and Refugees: A Systematic Review

**DOI:** 10.1007/s40615-024-02036-7

**Published:** 2024-05-28

**Authors:** Jingyeong Song, Jonathan Corcoran, Renee Zahnow

**Affiliations:** School of Social Science, The University of Queensland, Brisbane, QLD 4072 Australia

**Keywords:** Social Connectedness, Well-Being, Life Satisfaction, Resettlement, Refugees, Systematic Review

## Abstract

In the context of rising global migration and/or forced displacement, there is a pressing need to consider the well-being and life satisfaction of (im)migrants and refugees during resettlement. Research highlights the importance of social connectedness for (im)migrants and refugees during the resettlement phase. Yet, a critical gap remains in our understanding of the key characteristics through which social connections exert their influence on well-being and life satisfaction. This systematic review provides a comprehensive overview of the existing international literature on social connectedness and its impact on the well-being and life satisfaction of (im)migrants and refugees in post-migration contexts. Our analysis of 43 studies finds that social connectedness, in the form of social support and social networks, is beneficial for well-being and life satisfaction during resettlement. In addition to social support from individual network ties, community engagement can also enhance well-being by facilitating a sense of belonging. All 43 studies focused on psychological well-being and 69.8% relied on cross-sectional data. The findings of this review emphasise the need for longitudinal studies and standardised measurement tools to capture the dynamic interplay between social connectedness and well-being across various domains (psychological, physical, general/subjective) in migrant populations. We draw on the findings to propose a new conceptual model of the dynamic association between social connectedness and well-being/life satisfaction that seeks to explore these relationships in future empirical studies.

**Supplementary Information:**

The online version contains supplementary material available at 10.1007/s40615-024-02036-7.

## Introduction

The global landscape of human movement has undergone a significant transformation in recent decades, with an increasing number of individuals residing in countries other than their birthplaces. In 2024, the International Organization for Migration (IOM) estimated that 281 million people, 3.6% of the global population, were international migrants [1]. Motivations for migration vary significantly. Some individuals voluntarily move to improved opportunities, while others are driven from their country of origin by conflict or persecution. A significant portion of migration is driven by necessity. At the end of 2022, the United Nations Refugee Agency (UNHCR) estimated that over 35.3 million people were forcibly displaced as refugees [2]. For individuals in this context, resettlement can be a challenging experience. (Im)migrants and refugees often experience lower levels of well-being and life satisfaction compared to native-born residents in their host countries, and refugees are recognised as one of the most vulnerable groups in our society in terms of risk for poor health [3–5] While navigating the challenges of resettlement, rebuilding social connections presents a significant yet often overlooked obstacle. The absence of close relationships and a sense of belonging can lead to increased stress, isolation, and physical health problems, that hinder the resettlement process [6, 7].

The significance of social connectedness for health and well-being is well-established in the literature [8]. Recently, studies have increasingly focused on the evidence of health impacts, societal trends in social implications for specific groups like the aging population, and the influence of major events like pandemics [9]. Research with refugee samples in the context of resettlement further underscores this crucial role of social connection for individual physical health and psychological well-being [10–12]. Recognising its importance for both health and well-being, studies emphasise the need to foster a sense of community and belonging for refugees during resettlement [13]. Studies also demonstrate the deleterious impact of weak or absent social connections during resettlement [11]. Wei et al. [12] find that feeling connected to both mainstream and ethnic communities is associated with lower chronic loneliness, stress, anxiety, and depression, as well as higher self-esteem and life satisfaction. This suggests a dual protective effect of social connection, mitigating the negative impacts of isolation while fostering positive psychological well-being. Liao and Weng [14] further contribute to this understanding by highlighting that strong social connections are associated with increased social engagement, effective communication, and a positive outlook on life.

Despite the importance of social connectedness for (im)migrants and refugees in promoting well-being and life satisfaction during the resettlement phase, a critical gap remains in our understanding of the specific mechanisms through which these connections exert their influence. This systematic review aims to address this gap by comprehensively synthesising the existing international literature on social connectedness and well-being among (im)migrants and refugees’ populations in post-migration contexts. By analysing the diverse types of social connections and their nuanced relationships with well-being and life satisfaction, this study seeks to contribute to a more robust understanding of the multifaceted role social connectedness plays in the lives of (im)migrant and refugee populations. The findings of this systematic review will have significant implications for the development and implementation of evidence-based social support programs tailored to the specific challenges associated with resettlement for refugees.

## Methods

The study follows the guidelines and recommendations of the Preferred Reporting Items for Systematic Reviews and Meta-Analyses (PRISMA) 2020 [15].

### Eligibility Criteria

A set of inclusion and exclusion criteria was used. The inclusion criteria were as follows:

1. Published peer-reviewed articles
2. Published in the English language
3. Focused on population groups: immigrants, migrants, and refugees
4. Focused on adults (defined as those aged 18 years and above)
5. Focused on empirical studies (e.g. quantitative, qualitative, and mixed-methods)

Studies were excluded based on the following criteria: if the article was a narrative review, theory, or conceptual framework; or if it addressed specific topics such as COVID-19, heart disease, HIV, or disasters (e.g. floods, tsunamis, or any type of natural/man-made disasters).

### Information Sources and Search Strategy

We conducted systematic searches of three electronic databases (PubMed, SCOPUS, and Web of Science) using a combination of search terms to identify pertinent studies examining the influence of social connections on well-being and life satisfaction among refugees and (im)migrants. We conducted the searches between July 18 to 20, 2023 using a predefined search strategy: (Social ties OR Social tie OR Sense of belonging OR Sense of community) AND (Immigrant OR Immigrants OR Migrant OR Migrants OR Refugee OR Refugees) AND (Well-being OR Life satisfaction) AND (Resettlement). No restrictions were applied regarding the year of publication. Further information on the electronic search strategy is presented in the Supplementary Table S1.

### Selection Process and Data Extraction

**Fig. 1:**
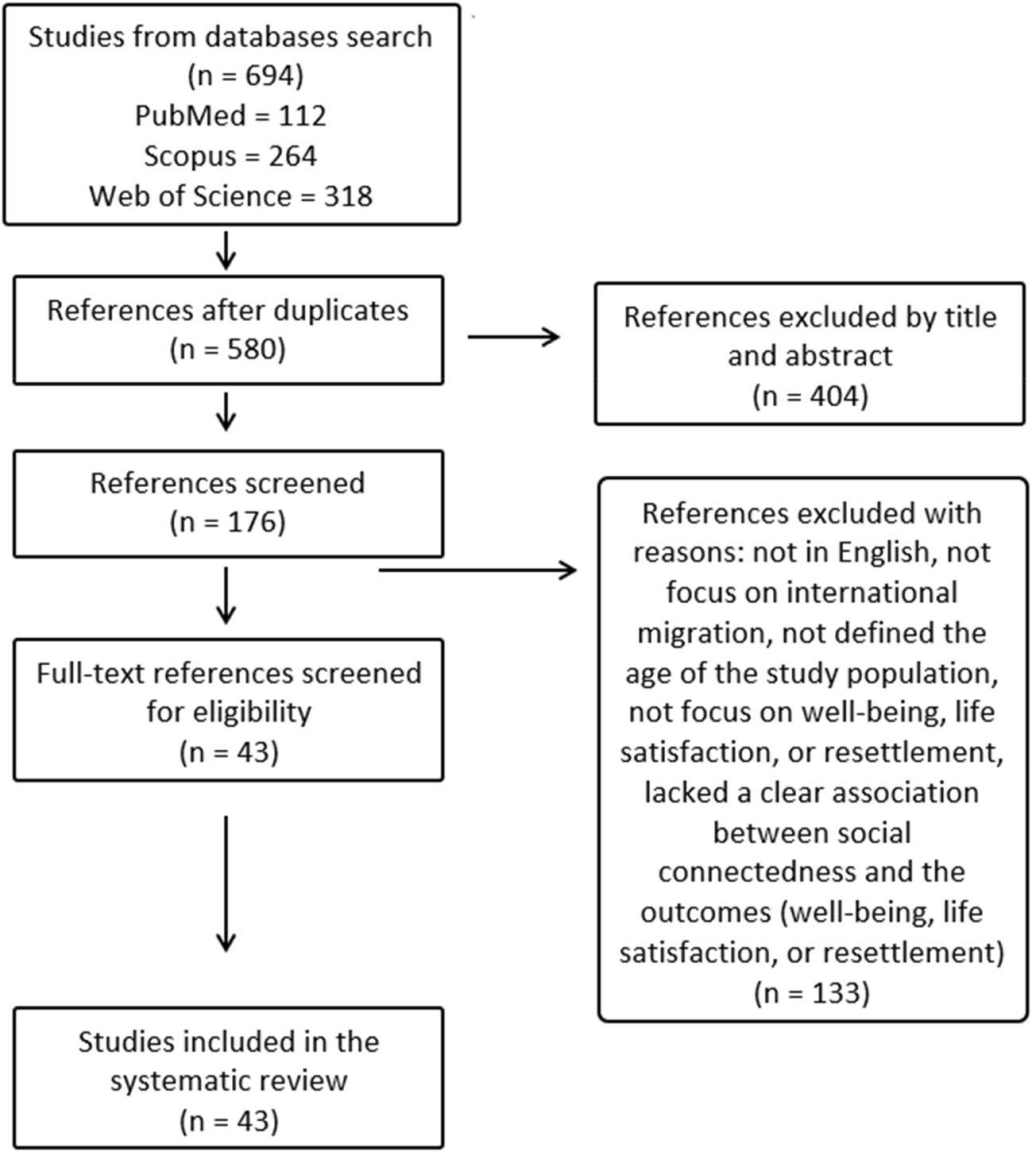
Systematic review process

Following the initial searches, which identified 694 eligible documents, we applied the inclusion/exclusion criteria to screen all titles and abstracts. This was conducted utilising the PRISMA software which enables reviewers to screen documents independently and highlights if mismatches occur. Twenty percent of documents were screened by two reviewers to ensure criteria were applied consistently. Agreement between reviewers was consistently high and all mismatches were resolved. A total of 176 studies qualified for full-text review, of which 43 met the final inclusion criteria (see Fig. 1). A full list of included studies is presented in Supplementary Table S2 and further details on the selection process, and data extraction procedures are also available in the supplementary materials.

## Results

### Methods and Study Design

The 43 studies comprising the final sample of eligible documents were published between 1999 and 2023 (see Table 1 and Supplementary Fig. S1). Beginning in 2018, there was a noticeable uptick in the number of studies addressing the concepts of social connectedness and immigrant well-being. Approximately, 80% (*n* = 34) of the studies were published in the 6 years between 2018 and 2023, reflecting increasing attention to refugees’ well-being and resettlement and its social determinants coinciding with the rise of the refugee crisis [16, 17]. A total of 34.9% of the included studies used quantitative methods, 39.5% adopted qualitative methods and 9.3% used mixed methods. The majority of studies apply a cross-sectional design (69.8%). Only 11.6% (*n* = 5) of all studies in our sample use longitudinal data. Of the five studies that apply a longitudinal design, on average the study follow-up period is 3.3 years. The longest study follow-up period was 6 years [18], while the shortest follow-up period was 6 months, with assessments occurring 3 to 5 months following the baseline assessment [19]. More detailed information is presented in Table 1.

**Table 1:** Author(s), year, methods, research design, sample collection, spatial scale, and type of publication

Author(s)	Year	Empirical method(s)	Cross-sectional /longitudinal	Sample collection	Spatial Scale	Type of publication
Abi Zeid Daou, K. R	2022	Systematic review	NA	NA	NA	Journal article
Al-Adhami, M., Berglund, E., Wångdahl, J., and Salari, R	2022	Quantitative (descriptive/modelling)	Cross-sectional	Civic orientation course	Countywide	Journal article
Alexander, N., Mathilde, S., and Øivind, S	2021	Quantitative (descriptive/modelling)	Cross-sectional	National	Nationwide	Journal article
Baeza-Rivera, M. J., Salazar-Fernández, C., Manríquez-Robles, D., Salinas-Oñate, N., and Smith-Castro, V	2022	Quantitative (descriptive/correlational/modeling)	Cross-sectional	Online survey	Regional	Journal article
Bedaso, A., and Duko, B	2022	Systematic review	NA	NA	NA	Journal article
Brailovskaia, J., Schönfeld, P., Kochetkov, Y., and Margraf, J	2019	Quantitative (descriptive/modelling)	Cross-sectional	National	Nationwide	Journal article
Campbell, M. R., Mann, K. D., Moffatt, S., Dave, M., and Pearce, M. S	2018	Quantitative (descriptive/modelling)	Cross-sessional and longitudinal	National	Nationwide	Journal article
Disney, L. R., McPherson, J., and Jamal, Z. S	2021	Qualitative (semi-structured/interview/thematic analysis)	Cross-sectional	Community-based	Statewide	Journal article
Dowling, A., Kunin, M., and Russell, G	2022	Qualitative (semi-structured interview/ phenomenological analysis)	Cross-sectional	Centres-based	Citywide	Journal article
Greene, R. N	2019	Mixed (interview/descriptive/modelling)	Longitudinal	Program-based/native-speaking interpreters	Citywide	Journal article
Hawkes, C., Norris, K., Joyce, J., and Paton, D	2021	Systematic review	NA	NA	NA	Journal article
Jankovic-Rankovic, J., Oka, R. C., Meyer, J. S., Snodgrass, J. J., Eick, G. N., and Gettler, L. T	2022	Mixed (semi-structured interview/ descriptive/MANOVA/modelling)	Cross-sectional	Asylum centres	Citywide	Journal article
Kahil, R., Iqbal, M., and Maghbouleh, N	2022	Qualitative (in-depth interview)	Longitudinal	Organizations-based	Citywide	Journal article
Kikhia, S., Gharib, G., Sauter, A., Vincens, N. C. L., and Loss, J	2021	Qualitative (in depth interview/ Content analysis)	Cross-sectional	Various channels; organisations, social media networks and professionals	Citywide	Journal article
Kindermann, D., Zeyher, V., Nagy, E., Brandenburg-Ceynowa, H., Junne, F., Friederich, H. C., Bozorgmehr, K., and Nikendei, C	2020	Quantitative (descriptive/wilcoxon signed-rank test/modelling)	Longitudinal	Clinic	Citywide	Journal article
King, R. U., Heinonen, T., Uwabor, M., and Adeleye-Olusae, A	2017	Qualitative (photovoice approach/interview/focus groups)	Cross-sectional	Various channels: flyers, Community organizations, church, and key stakeholders	Citywide	Journal article
Mahadevan, R., and Jayasinghe, M	2024	Quantitative (descriptive/modelling)	Longitudinal	National	Nationwide	Journal article
Martzoukou, K., and Burnett, S	2018	Qualitative (interview, focus group)	Cross-sectional	Community centres-based	Urban/rural	Journal article
Miller, R., Tomita, Y., Ong, K. I. C., Shibanuma, A., and Jimba, M	2019	Systematic review	NA	NA	NA	Journal article
Modesti, C., and Talamo, A	2021	Scoping review	NA	NA	NA	Journal article
Mwanri, L., Miller, E., Walsh, M., Baak, M., and Ziersch, A	2023	Qualitative (semi-structured interview/coding analysis)	Cross-sectional	Community-based	Rural town	Journal article
Nilsson, H., Saboonchi, F., Gustavsson, C., Malm, A., and Gottvall, M	2019	Qualitative (focus group/content analysis)	Cross-sectional	Swedish Red Cross Treatment Centre	Citywide	Journal article
Njororai, F., and Lee, S	2018	Quantitative (descriptive/*t*-test/correlational/modelling)	Cross-sectional	Community-based/translator	Citywide	Journal article
Remennick, L. I	1999	Qualitative (interview)	Cross-sectional	Local public hospitals	Citywide	Journal article
Remennick, L. I	2001	Qualitative (interview)	Cross-sectional	Hospitals	Citywide	Journal article
Ryan, D., Tornberg-Belanger, S. N., Perez, G., Maurer, S., Price, C., Rao, D., Chan, K. C. G., and Ornelas, I. J	2021	Quantitative (descriptive/modelling)	Cross-sectional	Community-based organizations	Countywide	Journal article
Sá, F. H. de L., Waikamp, V., Freitas, L. H. M., and Baeza, F. L. C	2022	Systematic review	NA	NA	NA	Journal article
Shahzeidi, M., Stone, G., and Filiz, B	2023	Quantitative (descriptive/modelling)	Cross-sectional	Online channels	Citywide	Journal article
Slade, N., and Borovnik, M	2018	Qualitative (in-depth interview/thematic analysis)	Cross-sectional	Red Cross Refugee Service	Citywide	Journal article
Sossou, M. A., Craig, C. D., Ogren, H., and Schnak, M	2008	Qualitative (interview/ narrative analysis)	Cross-sectional	Organization-based	Medium-urban	Journal article
Sulaiman-Hill, C. M. R., and Thompson, S. C	2012	Mixed (interview, descriptive)	Cross-sectional	Various channels: Afghan ethnic groups, religious affiliations, Community leaders	Citywide	Journal article
Sulaiman-Hill, C. M. R., and Thompson, S. C	2012	Mixed (interview/independence test/descriptive)	Cross-sectional	Various channels: Afghan ethnic groups, religious affiliations, Key individuals within the community	Citywide	Journal article
Suto, M. J	2013	Qualitative (semi-structured interview)	Cross-sectional	Community resettlement agency, services, and organization/advertisement	Not specified	Journal article
Tinghög, P., Malm, A., Arwidson, C., Sigvardsdotter, E., Lundin, A., and Saboonchi, F	2017	Quantitative (descriptive/modelling)	Cross-sectional	National	Nationwide	Journal article
Tip, L. K., Brown, R., Morrice, L., Collyer, M., and Easterbrook, M. J	2019	Quantitative (descriptive/modelling)	Longitudinal	National	Nationwide	Journal article
Tippens, J. A., Roselius, K., Padasas, I., Khalaf, G., Kohel, K., Mollard, E., and Sheikh, I	2021	Qualitative (photovoice project/ narrative analysis)	Cross-sectional	Church/classroom at university	Citywide	Journal article
Um, M. Y., Chi, I., Kim, H. J., Palinkas, L. A., and Kim, J. Y	2015	Quantitative (descriptive/modelling)	Cross-sectional	National	Nationwide	Journal article
Waardenburg, M., Visschers, M., Deelen, I., and van Liempt, I	2019	Qualitative (interview/coding analysis)	Cross-sectional	Reception centre	Municipality	Journal article
Wachter, K., Dalpe, J., Bonz, A., Drozdowski, H., and Hermer, J	2021	A scoping review	.NA	NA	NA	Journal article
Wachter, K., and Gulbas, L. E	2018	Qualitative (interview)	Cross-sectional	Resettlement agency	Mid-size town	Journal article
Wanna, C. P., Seehuus, M., Mazzulla, E., and Fondacaro, K	2019	Quantitative (descriptive/modelling)	Cross-sectional	Community mental health clinic	Citywide	Journal article
Yun, S., Ahmed, S. R., Hauson, A. O., and Al-Delaimy, W. K	2021	Quantitative (descriptive/modelling)	Cross-sectional	Organization-based	Citywide	Journal article
Ziersch, A., Miller, E., Baak, M., and Mwanri, L	2020	Qualitative (interviews/thematic analysis)	Cross-sectional	Community-based	Rural town	Journal article

### Sample Collection Spatial Scale

**Fig. 2:**
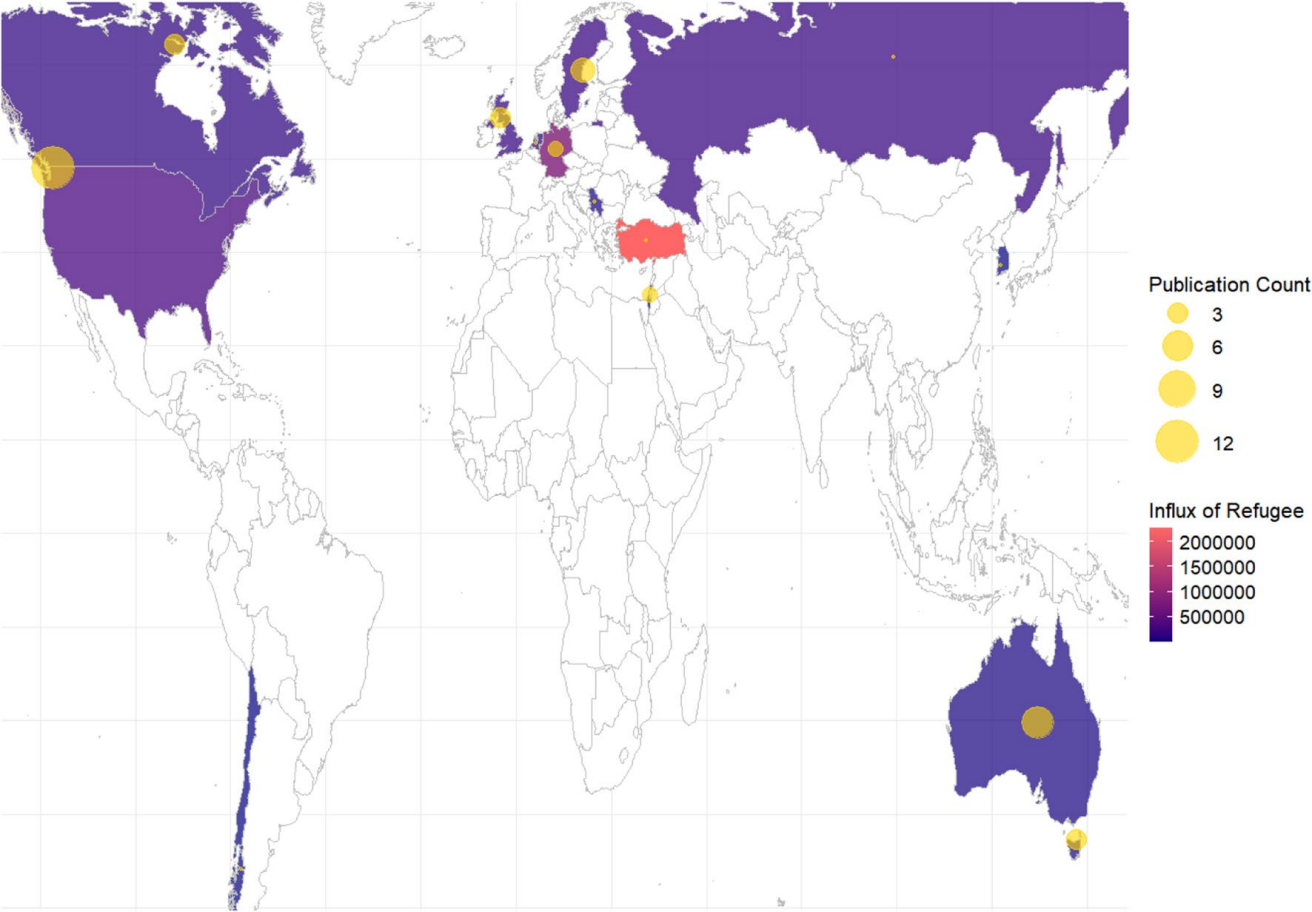
Mapping academic publications and refugee inflow in host countries. Figure 2 shows the countries where the research papers were published, represented by yellow bubbles indicating the number of papers. This map is overlaid with data on the average refugee influx into these countries from 2010 to 2022

Most of the studies (72.1%, *n* = 31) were conducted with samples of resettled individuals residing in community settings within Western countries (Australia, Canada, Germany, Netherlands, New Zealand, Scotland, Sweden, the UK, and the USA). Geographic hotspots of publication include the USA, Canada, Australia, the UK, Germany, Sweden, and the Netherlands, all of which have ratified the 1951 Refugee Convention and its 1967 protocol. Among these, the USA (*n* = 10) and Australia (*n* = 6) have the largest circles (see Fig. 2), reflecting a significant academic focus within these countries on the subject of refugees or (im)migrants’ health and resettlement issues. Eight studies were conducted in non-western countries (Chile, Israel, Japan, Russia, Serbia, South Korea, and Turkey). Turkey is the highest refugee influx (mean = 2,278,586) country among the final studies, while only one paper was published. It may suggest a potential disparity between the magnitude of refugee influx in Turkey and the volume of studies concentrated on refugee issues within the country that are published in mainstream academic journals.

The majority of studies were conducted in an individual city (*n* = 17). Seven studies had a national focus, while six were carried out at the state, county, regional, or municipality level. Five studies focused on either urban/rural or town-sized settings. Eight studies did not specify the spatial scale of focus. Approximately half (*n* = 21) of the studies recruited participants through third-party community organisation and/or utilised center-based samples. Other methods of participant recruitment occurred at hospitals (*n* = 4) or through online surveys and programs, including orientation courses (*n* = 5). Further information is available in Table 1.

### Participants’ Characteristics

Thirty-two of the 43 studies (74.4%) focused on refugees, and fewer studies (*n* = 9, 20.9%) focused on immigrants, migrants, and humanitarian migrants. Only two studies focused specifically on displaced people and asylum seekers. One of the eligible studies analysed differences between migrant and non-migrant groups. Across 40 studies, the sample sizes ranged from 2 to 5678 participants (*N* = 80,265), with the exclusion of three studies [20–22] as they reported only the number of articles, not participant counts. The sample sizes of the quantitative studies ranged from 50 to 5678 participants, with a total of 16,189 participants. On average, quantitative studies included 1079 participants. Qualitative study sample sizes ranged from 2 to 44, with a total of 362 participants. On average, qualitative studies included 19.1 participants. Mixed study sample sizes ranged from 76 to 290, with a total of 640 participants and the average sample size was 160 participants (see Table 2).

**Table 2:** Author(s), year, sample size, age, gender, population type, country of migration (host)

Author(s)	Year	Sample size	Age (mean (SD)/*n*/%/range)	Gender (female (*n*/%))	Population type	Country of migration (host)
Abi Zeid Daou, K. R	2022	350 (approximately)	NA	100%	Refugees	NA
Al-Adhami, M., Berglund, E., Wångdahl, J., and Salari, R	2022	787	19–29: 231 (31.3%) 30–49: 415 (56.2%) 50–69: 92 (12.5%)	436 (56%)	Refugee migrants	Sweden
Alexander, N., Mathilde, S., and Øivind, S	2021	1215	18–29:58.8 (26.5%) 30–39:59.2 (27.3%) 40–49:58.2(27.4%) > = 50: 31.1 (26.9%)	54.8 (26.4%)	Refugees	Sweden
Baeza-Rivera, M. J., Salazar-Fernández, C., Manríquez-Robles, D., Salinas-Oñate, N., and Smith-Castro, V	2022	283	34.4(9.53), (18–72)	189(67%)	Immigrants	Chile
Bedaso, A., and Duko, B	2022	53,458 (baseline)	> = 18	NA	Displaced people	NA
Brailovskaia, J., Schönfeld, P., Kochetkov, Y., and Margraf, J	2019	4635	Russian migrant: 43.19(14.43) Russian non-migrant:40.65(16.53) USA migrant:48.61(18.62) USA non-migrant:55.76 (17.27)	Russian migrant:49.5%, Russian non-migrant:53.9% USA migrant:59.5% USA non-migrant:60.6%	Migrant/non-migrant	Russia/USA
Campbell, M. R., Mann, K. D., Moffatt, S., Dave, M., and Pearce, M. S	2018	5678 (baseline)	18–24: 1249 (21.9%) 25–34: 2673 (46.9%) 35–44: 1079 (19%) 45–64: 450 (7.9%) 65 + : 108 (1.9%) Missing: 137 (2.4%)	36% (initial survey)	Refugees	UK
Disney, L. R., McPherson, J., and Jamal, Z. S	2021	12	43 (29–61)	6	Refugees	USA
Dowling, A., Kunin, M., and Russell, G	2022	19	20–29: 7 (37%) 30–39: 6 (32%) 40–49: 4 (21%) 50–59: 2 (10%)	15 (79%)	Refugees	Australia
Greene, R. N	2019	290	34.6 (11.53)	52%	Refugees	USA
Hawkes, C., Norris, K., Joyce, J., and Paton, D	2021	600	NA	NA	Refugees	NA
Jankovic-Rankovic, J., Oka, R. C., Meyer, J. S., Snodgrass, J. J., Eick, G. N., and Gettler, L. T	2022	76	30.14(7.75)	35	Refugees	Serbia
Kahil, R., Iqbal, M., and Maghbouleh, N	2022	2	> = 60	2 (100%)	Refugees	Canada
Kikhia, S., Gharib, G., Sauter, A., Vincens, N. C. L., and Loss, J	2021	9	40 (18–55)	9 (100%)	Immigrants	Germany
Kindermann, D., Zeyher, V., Nagy, E., Brandenburg-Ceynowa, H., Junne, F., Friederich, H. C., Bozorgmehr, K., and Nikendei, C	2020	84	32 (8.7)	33 (39.3%)	Asylum seekers	Germany
King, R. U., Heinonen, T., Uwabor, M., and Adeleye-Olusae, A	2017	15	21–57	8	Refugees	Canada
Mahadevan, R., and Jayasinghe, M	2024	1,058	Wave 1: 36.75 (13.4) Wave 5: 40.18 (14.11)	Wave1: 44.8% Wave5: 47.1%	Humanitarian migrants	Australia
Martzoukou, K., and Burnett, S	2018	39	18–27:5 28–37:3 38–47:4 48–57:4 58–64:2 Undisclosed:2	21	Refugees	Scotland
Miller, R., Tomita, Y., Ong, K. I. C., Shibanuma, A., and Jimba, M	2019	8,649	NA	NA	Migrants	Japan
Modesti, C., and Talamo, A	2021	18 (articles)	NA	NA	Refugees	NA
Mwanri, L., Miller, E., Walsh, M., Baak, M., and Ziersch, A	2023	44	18–68	NA	Refugees	Australia
Nilsson, H., Saboonchi, F., Gustavsson, C., Malm, A., and Gottvall, M	2019	33	45	10	Refugees	Sweden
Njororai, F., and Lee, S	2018	50	43.6(16.6)	31 (62%)	Refugees	USA
Remennick, L. I	1999	10	35–39: 23% 40–44: 27% 45–49: 36% 50–55:14%	10 (100%)	Immigrants	Israel
Remennick, L. I	2001	20	49	20 (100%)	Immigrants	Israel
Ryan, D., Tornberg-Belanger, S. N., Perez, G., Maurer, S., Price, C., Rao, D., Chan, K. C. G., and Ornelas, I. J	2021	153	40.2(10.2)	153 (100%)	Immigrants	USA
Sá, F. H. de L., Waikamp, V., Freitas, L. H. M., and Baeza, F. L. C	2022	64 (articles)	NA	NA	Refugees	NA
Shahzeidi, M., Stone, G., and Filiz, B	2023	203	19–24: 33 (16.3%) 25–34: 55 (27.1%) 35–44: 63 (31%) 45–54: 19 (9.4%) 55–64:7 (3.4%) 65 + :5 (2.5%) No answer: 21 (1.3%)	81 (41.4%)	Refugees	Turkey
Slade, N., and Borovnik, M	2018	3	57,59,78	2	Refugees	New Zealand
Sossou, M. A., Craig, C. D., Ogren, H., and Schnak, M	2008	7	32–47	7 (100%)	Refugees	USA
Sulaiman-Hill, C. M. R., and Thompson, S. C	2012	193	18–70	93	Refugees	Australia /New Zealand
Sulaiman-Hill, C. M. R., and Thompson, S. C	2012	81	Under 30: 30 (37%) 30 and over: 51 (63%)	36 (44%)	Refugees	Australia /New Zealand
Suto, M. J	2013	14	20–55	14 (100%)	Immigrants	Canada
Tinghög, P., Malm, A., Arwidson, C., Sigvardsdotter, E., Lundin, A., and Saboonchi, F	2017	1,215	18–29: 23.3% 30–39:32.9% 40–49: 24.3% 50–64: 19.5%	37.20%	Refugees	Sweden
Tip, L. K., Brown, R., Morrice, L., Collyer, M., and Easterbrook, M. J	2019	180	37.2 (18–80)	84	Refugees	UK
Tippens, J. A., Roselius, K., Padasas, I., Khalaf, G., Kohel, K., Mollard, E., and Sheikh, I	2021	9	> = 19	9 (100%)	Refugees	USA
Um, M. Y., Chi, I., Kim, H. J., Palinkas, L. A., and Kim, J. Y	2015	261	41.1(9.11)	64%	Refugees	South Korea
Waardenburg, M., Visschers, M., Deelen, I., and van Liempt, I	2019	18	18–26	4	Refugees	Netherlands
Wachter, K., Dalpe, J., Bonz, A., Drozdowski, H., and Hermer, J	2021	20 (articles)	NA	NA	Refugees	NA
Wachter, K., and Gulbas, L. E	2018	27	18–29: 6 30–39: 14 > 40: 7	27 (100%)	Refugees	USA
Wanna, C. P., Seehuus, M., Mazzulla, E., and Fondacaro, K	2019	179	42.4 (13.1)	51.70%	Refugees	USA
Yun, S., Ahmed, S. R., Hauson, A. O., and Al-Delaimy, W. K	2021	219	38.79 (6.87)	219 (100%)	Refugees	USA
Ziersch, A., Miller, E., Baak, M., and Mwanri, L	2020	44	< 25: 4 26–50: 27 51–68: 9 Unspecified: 4	22	Refugees	Australia

Of the total 43 studies, 15 reported details of participants’ average age and associated standard deviation. Nine studies (21%) reported the frequency and/or percentage of the sample within age categories. Three studies reported both average age and frequency and/or percentage of the age categories. Five studies reported the age range of the sample. Other studies simply reported the participants were above a certain age. For example, two studies reported participants were 18 years and over [23, 24]. One study recruited only elders aged 60 years and older [18]. The majority of studies (58.1%, *n* = 25) recruited both male and female participants. Of the total 43 studies, 25.6%, (*n* = 11) used female-only samples (see Table 2).

### Well-Being Outcomes

**Fig. 3:**
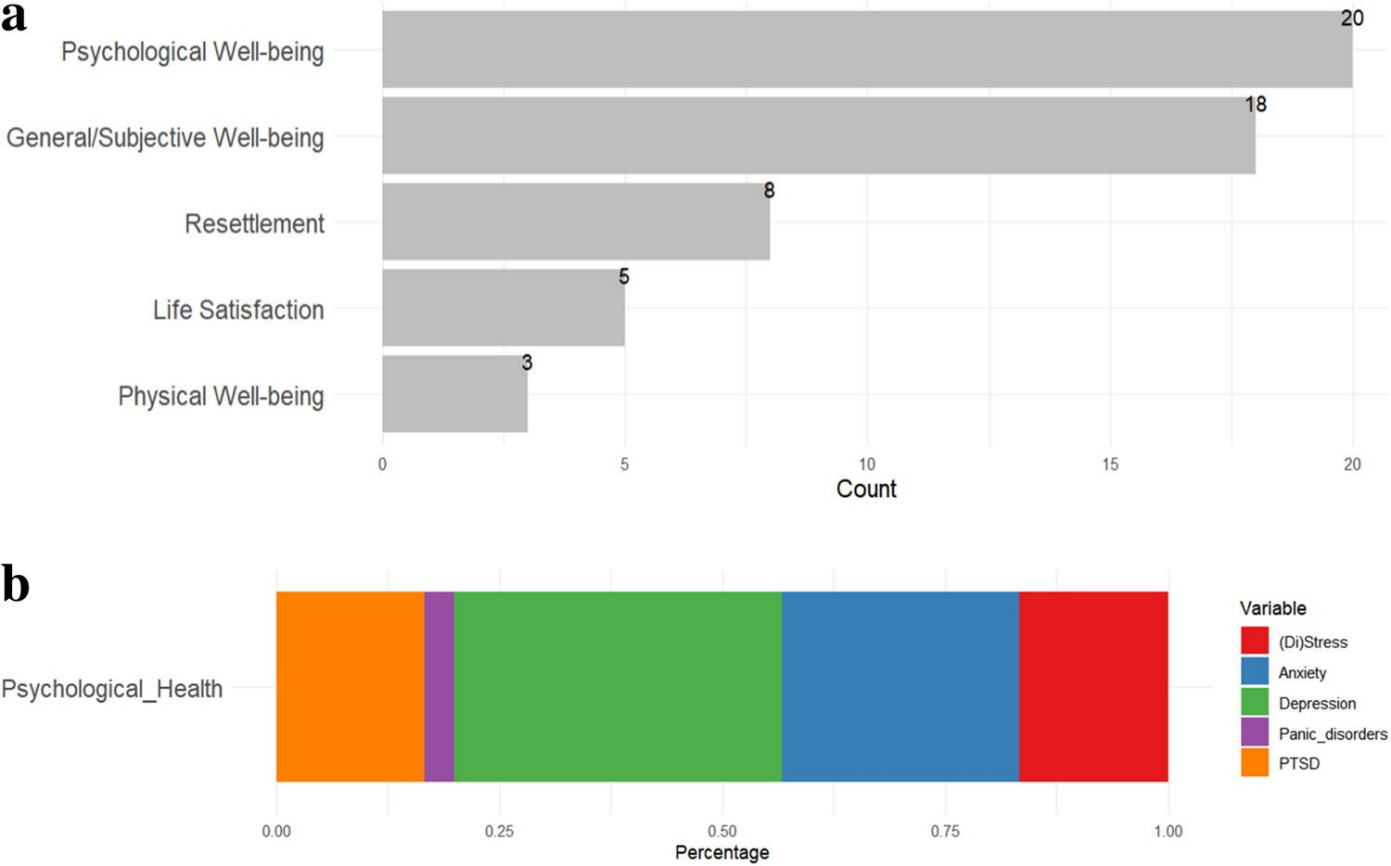
**a** Bar plot for type of outcomes (overall). **b** Bar plot for psychological well-being (in detail)

Well-being was conceptualised and measured differently across the 43 studies. Most common studies focused on psychological well-being (*n* = 20, 46.5%), which included depression, anxiety, PTSD, and panic disorders (Fig. 3b). General well-being (*n* = 18, 41.9%) was the second most common conceptualisation of well-being that appeared among the sample. Eight studies focused on resettlement (18.6%), five on life satisfaction (11.6%), and three on physical well-being (7%), see Fig. 3a.

Seventy percent of the studies that used psychological well-being were measured within a medical framework, primarily through standardised clinical survey instruments such as the Hopkins Symptom Checklist (HSCL-25) [25–28]. The HSCL-25, a symptom inventory focused specifically on anxiety and depression, comprises 25 items divided into two parts: 10 assessing anxiety symptoms and 15 assessing depression symptoms. Each question offers four response options for participants to select. Notably, a significant portion of these items inquire about participants' feelings, including fear, nervousness, tension, restlessness, hopelessness, and worthlessness. Beyond the HSCL-25, other common tools for measuring psychological well-being include the Harvard Trauma Questionnaire (HTQ) for assessing traumatic stress [26, 27] and the Patient Health Questionnaire (PHQ) for depression or panic disorder [19, 29]. For general/subjective well-being, subjective questions were used, such as assessments of overall health status [30] and experiences in managing health in immigrant contexts [31]. The World Health Organization's Well-Being Index (WHO-5) was also employed in two studies [26, 32]. Resettlement and life satisfaction were similarly measured with subjective questions. Some studies measured overall resettlement experiences and life satisfaction [33], while others explored the impact of employment on life satisfaction and the resettlement experience [34]. Additionally, two studies [25, 35] utilised the World Health Organization Quality of Life assessment (WHOQOL) and the WHOQOL-BREF. For further details on how other concepts were measured, see Supplementary Table 3.

### Social Connectedness

**Fig. 4:**
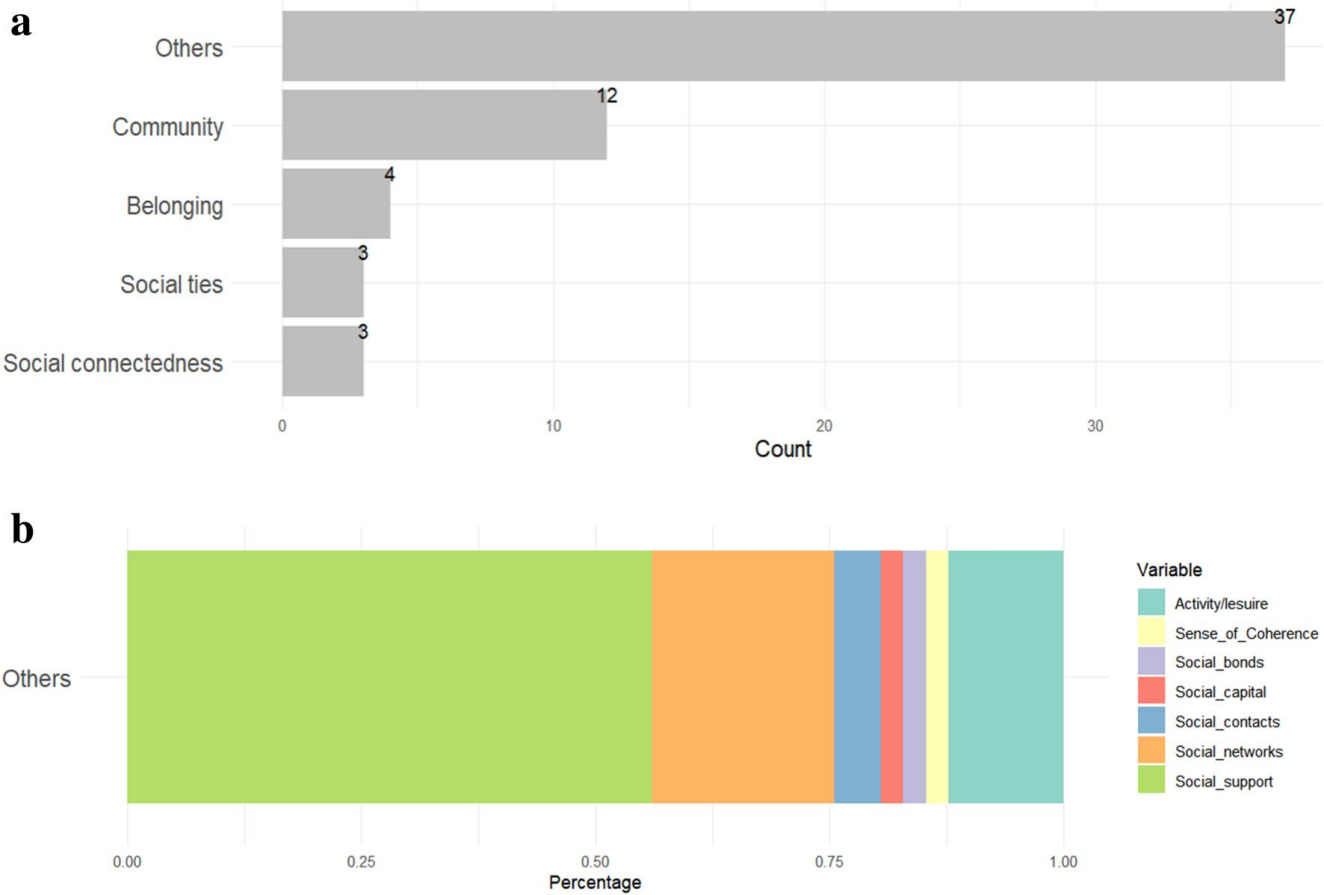
**a** Bar plot for social connectedness (overall). **b** Bar plot for social connectedness (in detail)

We included three keywords in the search terms to capture social connectedness. These were social ties, belonging, and community. However, the final sample of eligible documents also includes references to related terms including social support, social networks, social contacts, social capital, social bonds, and sense of coherence (*n* = 37). This highlights the complexity of social connection, which most studies seem to acknowledge by employing multiple measures. These measures range from main concepts such as social support, social networks, or social capital, derived from theoretical frameworks, to more specific sub-components like social contacts or social bonds. Such diversity in measurement reflects the multifaceted nature of social connectedness research and also underscores the challenge of finding a universally accepted singular measure. The majority of studies (*n* = 23, 53.5%) focused on social support, encompassing emotional, practical, and perceived support, alongside family, friends, community, and institutional support. In another focus area, 12 of the studies (28%) explored the concept of community, including community activity, host-community, and co-ethnic community engagement (see Fig. 4a, b).

In terms of social connectedness measurement tools, social support emerged as the most frequently measured aspect of social connectedness. Researchers assessed it primarily through subjective questions, often asking participants how often they seek help, talk to someone, or share their feelings with others [16, 25, 29, 30, 36–38]. Additionally, some studies employed validated instruments like the 5-item ENRICHD Social Support Instrument and the F-SozU K-14 [32, 39]. For instance, Ryan et al. [29] utilised an abbreviated version of the Medical Outcomes Study Social Support Measure. Further detailed information is presented in Supplementary Table S3.

### Associations Between Social Connectedness and Well-Being and Life Satisfaction

Of the 43 studies, 23 studies (53.5%) focused on social supports and overall outcomes associated with well-being and life satisfaction/resettlement. This was the most commonly examined relationship between the social connectedness indicators identified in this systematic review. Of 23 studies, 61% examined the impact of social support/networks on psychological well-being as the outcome variable. Meanwhile, 30.4% focused on general/subjective well-being and resettlement outcomes respectively. Three studies concentrated on life satisfaction and only one study focused on physical well-being.

The overall findings indicate that a lack of social support, encompassing emotional, practical, and relational aspects, is closely associated with adverse mental health outcomes, including depression, stress, and PTSD [21, 30, 31, 37, 40–42]; general/subjective well-being [26, 30]; and life satisfaction [43]. In contrast, strong social supports—comprising family, friends, community groups, and government support—often mitigate these negative impacts on psychological well-being [21, 23, 39, 44]; general/subjective well-being [21]; resettlement process [18, 22, 45, 46]. However, the influence of social support can be indirect or inconsistent [29, 32, 36, 47]. For example, Tinghög et al. [26] found that lack of family social support had the weakest association with depression and PTSD but was not significantly associated with anxiety. Similarly, Mahadevan and Jayasinghe [33] reported that ethnic support negatively correlated with life satisfaction in the male cohort compared to females. These findings highlight the importance of a supportive social environment in enhancing well-being and life satisfaction during the resettlement process, demonstrating its positive association with both well-being and life satisfaction. However, the findings also revealed the variations in the influence of these variables depending on the specific type of social support and the gender. Further detailed information is presented in Supplementary Table S4.

**Fig. 5:**
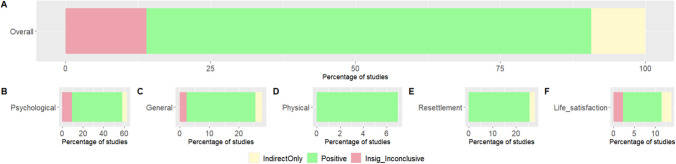
Bar plot for illustrating the association between social connectedness and outcomes

Figure 5 presents the empirical relationships identified in each study as positive, negative, indirect effect only, or insignificant/inconclusive. This classification highlights the connections between social connectedness (like social ties, community, belonging, and others) and the outcomes, providing an overview as well as specific insights for each of the five outcomes. Figure 5A displays the associations between social connectedness and the overall outcome across 43 studies. Most relationships were positive (76.7%), followed by insignificant/inconclusive (14%), and only indirect effects (9.3%). Figure 5B–F detail the associations between social connectedness and specific outcomes: psychological well-being (Fig. 5B), general/subjective well-being (Fig. 5C), physical well-being (Fig. 5D), resettlement (Fig. 5E), and life satisfaction (Fig. 5F). Overall, a positive correlation with each outcome is predominant.

Notably, there are no negative relationships, with the key concepts either having an indirect impact or showing varying results depending on the nature of the key concepts (for more details on the data used to generate Fig. 5 and Supplementary Table S4). For instance, Baeza-Rivera et al. [36] revealed an association between perceived social support and acculturative stress, reducing symptoms of mental health issues. Similarly, Disney et al. [34] identified a connection between the development of supportive networks associated with employment, improving life satisfaction, and resettlement experience. Regarding inconclusive findings, Greene’s study [25] showed that family ties were not associated with emotional distress but positively influenced psychological quality of life. Additionally, while family and friendship ties were not associated with emotional distress, ties with social service providers were positively associated with it. Additionally, Ryan et al. [29] observed that social support received through positive social interactions was associated with reduced anxiety symptoms. However, social ties, as well as emotional and affectionate support, were not found to be significant.

## Discussion

This systematic review sought to understand the empirical evidence on the association between social connectedness, well-being (e.g. psychological, physical, and general/subjective), and life satisfaction among refugees and (im)migrants during resettlement. The review focused on identifying the impact of social ties, a sense of belonging, community, and other social determinants like social support and networks on well-being, life satisfaction, and resettlement. Findings from a total of 43 studies demonstrate that research has largely focused on social support, particularly perceived emotional support, in relation to (im)migrant and refugees’ psychological well-being. Studies primarily assess subjective and objective emotional support rather than structural support and less attention has been given to other aspects of social connectedness such as community engagement and ethnic community connections. The findings consistently show that social support, both perceived and received, serves as a protective factor for resettlement-related well-being and life satisfaction. The benefits of social support for mental health and well-being outside of refugee populations are well-documented. Studies demonstrate social support provides a buffer against psychological distress and various disorders [48, 49]. In addition to social support, 28.3% of the studies in the review noted building and strengthening community networks was beneficial for psychological well-being, life satisfaction, and resettlement [20, 27, 34, 45, 47, 50, 51]. Notably, Remennick’s work [52, 53] and Tippens et al. [24] demonstrate how support from co-ethnic communities significantly boosts the psychological well-being and health of older women.

Findings related to two aspects of social connectedness; belonging and social ties, were less consistent across the 43 studies. Sense of belonging consistently emerged as a positive factor, with a sense of belonging linked to better settlement experiences, higher life satisfaction [33], and improved psychological well-being [46, 54]. However, it was only analysed in two studies in our sample. Alternatively, findings regarding the link between social ties and well-being were mixed. For example, Mwanri et al. [55] found that stronger social ties can boost subjective well-being, while Ryan et al. [29] concluded that there was no relationship between social ties and psychological well-being. These findings highlight the importance of considering the type of social ties when exploring their impact on well-being and life satisfaction. Different social connections may play distinct roles in shaping well-being. This finding demonstrates the need for more nuanced research to unpack the extent to which different forms of social ties influence well-being and to understand the different mechanisms through which bridging, bonding, and linking ties provide well-being benefits. Other aspects of social connectedness, such as participation in physical activities like sports and leisure, were positively associated with psychological well-being, physical well-being, and life satisfaction [35, 56–58]. Engaging in such activities and leisure can enhance community participation and meaningful social activities, address practical and emotional needs, and foster a sense of connectedness and belonging. Despite the growing evidence linking social connectedness to well-being and health outcomes for (im)migrants and refugees, we propose four key challenges that obstruct a nuanced understanding of its relationship with well-being and life satisfaction.

### Limited Scope of Well-Being

While a large number of studies focused on resettlement consider psychological well-being among immigrants and refugees during this period, other dimensions of well-being including general/subjective, physical well-being, and life satisfaction, remain relatively understudied. Among the limited studies that have focused on general/subjective, physical well-being, and life satisfaction, there is a lack of uniformity in measurement methods which makes comparison across studies difficult. A further limitation of current research is that well-being is almost universally conceptualised within a Western medicalised framework. For example, studies on psychological well-being commonly employ objective psychological health assessments utilised in clinical settings. This raises concerns about the applicability of conventional tools and scales, developed within this framework, to fully capture the multifaceted concept of well-being and its variations among (im)migrant and refugee populations [59]. For example, in certain cultures, such as Muslim communities, concepts of well-being may extend beyond Western medical considerations, encompassing dimensions of faith, religious practice, and spirituality [60, 61].

### Conceptual Ambiguity

The broad nature of social connectedness and its operationalisation under various terms and measures complicate comparisons and limit the effectiveness of policy interventions. Standardised measurement tools and a more nuanced understanding of different sub-categories within social connectedness, such as family, friends, and community ties, are essential for more precise research and policy development.

### Demographic and Socio-Cultural Factors

The impact of social support on well-being varies across demographic and socio-cultural groups, yet research in this area remains limited. While some studies explored the moderating effects of gender and age, studies remain limited in this area. Notably, no studies exclusively targeted interventions on male well-being and life satisfaction, while 11 studies focused solely on females. Only one study examined the influence of social support on life satisfaction and how this varied by gender [33]. Two studies revealed language proficiency as a potential moderator, noting fluency in English and active English language learning were associated with increased community engagement and in turn more positive health and resettlement outcomes for refugees [27, 47]. Only one study focused on the importance of social networks for spiritual support in addition to practical help. This study found that religious support facilitated greater access to services and resources which helped individuals to better navigate resettlement challenges [46]. Finally, socio-economic factors were identified as potential moderators, with social support acting to modify the association between financial strain and subjective well-being [32], alongside community networks as an important source of employment opportunities for refugees [34, 55]. In sum, there exists a need to evolve a more comprehensive understanding of well-being and life satisfaction in the post-migration process wherein future studies need to more fully consider factors like gender, ethnicity, cultural background, and their intersection, alongside social connectedness.

### Longitudinal Perspective

Only five studies employed a longitudinal research design, indicating a greater need to develop research that captures different aspects of social connectedness, such as social ties, support networks, belonging, and community involvement, alongside measures of well-being, throughout the resettlement period. Consistent with the findings of cross-sectional studies, a dominant pattern of results evident in the longitudinal analyses highlights a positive association between social connectedness and well-being/life satisfaction during resettlement. Notably, a larger proportion of the longitudinal studies, compared to the cross-sectional studies, reported inconclusive/insignificant findings regarding the association between social connectedness and both well-being and life satisfaction. This may suggest that the association between social connectedness and well-being among migrant populations is dynamic and fluctuates over time. Consequently, we argue that longitudinal research is crucial to understanding how social connectedness and well-being co-vary throughout the resettlement process, to evaluate the effectiveness of intervention, and to inform more effective and adaptive support programs tailored to individual needs. By addressing these challenges, future research can significantly improve our understanding of the complex relationship between social connectedness and well-being and life satisfaction for (im)migrants and refugees. This knowledge will inform the development of tailored social and economic support programs, ultimately contributing to a more successful and sustainable resettlement process.

### Publication Bias

This study focused on interventions targeting internationally mobile populations, including migrants, immigrants, and refugees. While the well-being and life satisfaction of internally displaced populations and refugees are critical social issues, the scope of this review was limited to examining interventions aimed at international populations. This study also excluded interventions targeting children; thus, insights regarding participants under the age of 18 are missing, as is the impact of children’s presence within households of adult refugees and immigrants on well-being and life satisfaction. This area requires further dedicated research and therefore falls outside the purview of this review. Additionally, studies in non-English languages were excluded but warrant further exploration, particularly in countries with high refugee populations and non-English dominant cultures, given their potential to provide valuable new insights.

## Conclusions

Resettlement into a new country presents significant challenges for (im)migrants and refugees, with implications for well-being and life satisfaction. Our systematic review highlights the potential for social connectedness to mitigate these challenges and foster positive outcomes. While the link between social connectedness and well-being/life satisfaction for (im)migrants and refugees is undeniable, a crucial gap remains in understanding the mechanisms behind it. This review delves into international research to bridge this gap, revealing that social support, particularly perceived emotional support, acts as a protective shield for well-being across diverse populations and settings. Engaging with communities and participating in activities positively influence both psychological and physical well-being, fostering a sense of belonging and integration. Belonging itself consistently emerges as a positive factor, linked to better settlement experiences, life satisfaction, and psychological well-being. However, the impact of social ties appears more nuanced, with some studies suggesting positive associations and others showing mixed results, calling for further research to unpack the specific roles of different types of social ties.

**Fig. 6:**
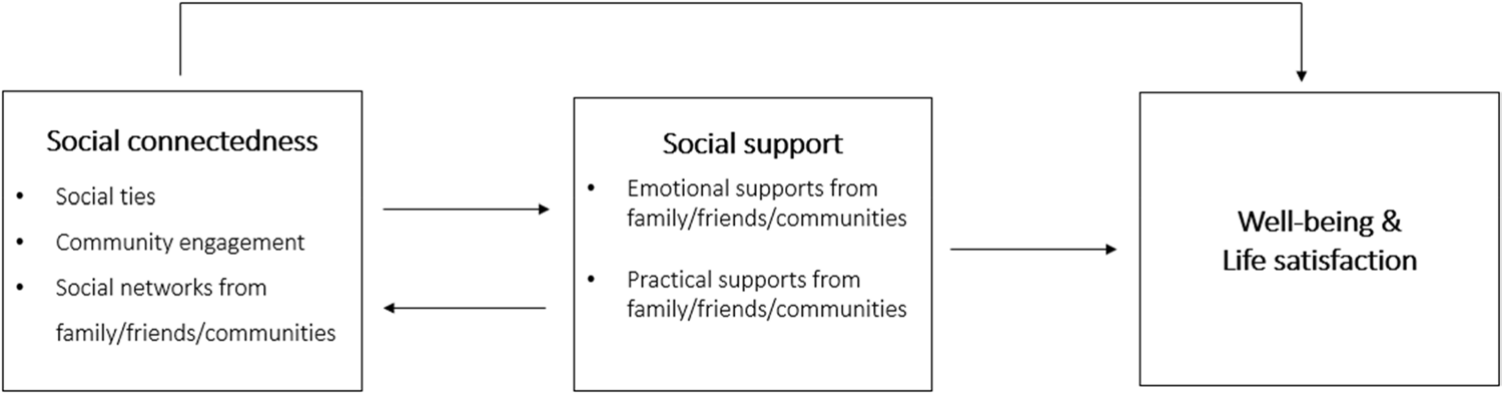
A proposed conceptual model

This systematic review contributes a comprehensive overview of existing literature, highlighting the importance of considering diverse types of social connections and their intricate relationships with well-being and life satisfaction. It also identifies critical knowledge gaps regarding the specific mechanisms underlying these connections and the moderating effects of individual characteristics. This knowledge paves the way for tailored social and economic support programs that address the specific needs of (im)migrant and refugee populations, ultimately leading to a sustainable resettlement process. Future research should delve deeper into the mechanisms through which social connections influence well-being, considering how different types of connections contribute to well-being through support, belonging, and identity formation, the role of social support in buffering against stressors and promoting resilience during resettlement, and the moderating effects of individual characteristics on these relationships. We underscore the need to move beyond a narrow focus on social support and embrace a broader understanding of social connectedness encompassing structural support, community engagement, and social intervention. Such an approach would enable the development of more comprehensive and effective support programs tailored to the specific needs of (im)migrant and refugee populations. Drawing on the findings from this review we propose a conceptual model (Fig. 6) that seeks to examine the dynamic association between social connectedness and well-being/life satisfaction. Our hope this that this model will help to orient future studies in terms of testing in empirical studies.

To support the development of effective intervention and support programs requires a shift towards longitudinal research designs that can assess the relationship between interventions aimed at strengthening social connectedness and long-term improvements in well-being and life satisfaction. Current refugee assistance programs, often characterised by short durations (12–24 months) and limited scope, exemplify the need for a more sustainable and holistic approach [62]. By building social connectedness, a strong community alongside government support, we can create a more effective and responsive network for identifying and addressing the specific needs of individuals navigating the complexities of resettlement. Ultimately, a deeper understanding of the diverse facets of social connectedness and their impact on well-being and life satisfaction will pave the way for the development of evidence-based interventions that empower (im)migrants and refugees to thrive in their new communities.

## Supplementary Information

Below is the link to the electronic supplementary material.

Supplementary file1 (DOCX 90 KB)

## Data Availability

Data and code are provided.
