# Supplementary material for: The Resettlement Journey: Understanding The Role of Social Connectedness on Well-being and Life Satisfaction among (Im)migrants and Refugees: A Systematic Review
Source: J Racial Ethn Health Disparities. 2024 May 28;12(4):2128–44. doi: 10.1007/s40615-024-02036-7 (PMC12241138; doi:10.1007/s40615-024-02036-7)

**Supplementary materials**

**Note**

1. Selection process and data extraction

A single author (J.S.) conducted the initial search, while two reviewers (J.S. and R.Z.) conducted the screening process. Both J.S. and R.Z. independently performed each step of the screening, from title/abstract to full-text article reviews. From the primary search, a total of 694 references were obtained, of which 114 were duplicates. J.S. screened the remaining 580 references, applying the eligibility criteria to their titles and abstracts. To ensure reliability, R.Z. independently screened 20% of these references. The agreement rate between the two reviewers was consistently high. Any disagreements were resolved through discussion, which subsequently led to the inclusion of additional criteria. For inclusion, studies needed to: focus on international migration; and involve populations living within refugee camps offering long-term or permanent settlement. A full-text review was conducted for 176 articles.130 articles were excluded for various reasons, such as: including populations of youth or adolescents under 18 years of age; not focusing on outcomes such as well-being, life satisfaction, or resettlement; or lacking a clear association between key concepts (social connections) and the outcomes. To minimise bias, R.Z. also independently reviewed 20% of the 176 full-text articles. Ultimately, 43 papers were identified for the final set. Data was extracted by JS and charted in Microsoft Excel. A key extraction sheet was developed including: the study characteristics (e.g., author’s name, year of publication, research design), participant characteristics (e.g., country of migration (host), population type, sample, gender, age), study setting (e.g., sample collection, spatial scale), measurement of key concepts and outcomes, and key findings.

**Table 1.** Electronic search strategy

PubMed:112

((((((((((((((("Social ties") OR ("Social tie")) OR ("Sense of belonging"))) OR ("Sense of community")) AND ("Immigrant"[Title/Abstract])) OR ("Immigrants"[Title/Abstract])) OR (Refugee[Title/Abstract])) OR (Refugees[Title/Abstract])) OR (Migrant[Title/Abstract])) OR (Migrants[Title/Abstract])) AND ("Life satisfaction")) OR ("Well-being")) ) AND ("Resettlement"))

Web of Science:318

(((((((((((((( ALL=( "Social ties" )) OR ALL=( "Social tie" )) OR ALL=("Sense of belonging" )) OR ALL=("Sense of community" )) ) AND TS=("Immigrant")) OR TS=("Immigrants") OR TS=("Refugees") OR TS=("Migrants") OR TS=("Refugee")) OR TS=( "Migrant"))) AND ALL=("Life satisfaction")) OR ALL=("Well-being"))) AND ALL=("Resettlement")))

Scopus: 264

ALL ( "Social ties"  OR  "Social tie"  OR  "Sense of belonging"  OR  "Sense of community" )  AND  TITLEABS ( "Immigrant"  OR  "Immigrants"  OR  "Refugees"  OR  "Migrants"  OR  "Refugee"  OR  "Migrant" )  AND  ALL ( "Life satisfaction"  OR  "Well-being" )  AND  ALL ( "Resettlement" )  AND  ( LIMIT-TO ( LANGUAGE ,  "English" ) )

**Table 2.** Full list of included studies in this review

| **Author(s)** | **Year** | **Citation** |  |  |
| --- | --- | --- | --- | --- |
| Abi Zeid Daou, K. R. | 2022 | Abi Zeid Daou, K. R. (2022). Refugee Mothers Mental Health and Social Support Needs: A Systematic Review of Interventions for Refugee Mothers. *Europe's Journal of Psychology*, *18*(3), 337-349. |  |  |
| Al-Adhami, M., Berglund, E., Wångdahl, J., & Salari, R. | 2022 | Al-Adhami, M., Berglund, E., Wångdahl, J., & Salari, R. (2022). A cross-sectional study of health and wellbeing among newly settled refugee migrants in Sweden-The role of health literacy, social support and self-efficacy. *PLoS ONE*, *17*, e0279397. |  |  |
| Alexander, N., Mathilde, S., & Øivind, S. | 2021 | Alexander, N., Mathilde, S., & Øivind, S. (2021). Post-migration Stressors and Subjective Well-Being in Adult Syrian Refugees Resettled in Sweden: A Gender Perspective. *Front in Public Health*, *9*. |  |  |
| Baeza-Rivera, M. J., Salazar-Fernández, C., Manríquez-Robles, D., Salinas-Oñate, N., & Smith-Castro, V. | 2022 | Baeza-Rivera, M. J., Salazar-Fernández, C., Manríquez-Robles, D., Salinas-Oñate, N., & Smith-Castro, V. (2022). Acculturative Stress, Perceived Social Support, and Mental Health: The Mediating Effect of Negative Emotions Associated with Discrimination. *International Journal of Environmental Research and Public Health*, *19*(24), 16522. |  |  |
| Bedaso, A., & Duko, B. | 2022 | Bedaso, A., & Duko, B. (2022). Epidemiology of depression among displaced people: A systematic review and meta-analysis. *Psychiatry Research*, *311*, 114493. |  |  |
| Brailovskaia, J., Schönfeld, P., Kochetkov, Y., & Margraf, J. | 2019 | Brailovskaia, J., Schönfeld, P., Kochetkov, Y., & Margraf, J. (2019). What Does Migration Mean to Us? USA and Russia: Relationship Between Migration, Resilience, Social Support, Happiness, Life Satisfaction, Depression, Anxiety and Stress. *Current Psychology*, *38*(2), 421-431. |  |  |
| Campbell, M. R., Mann, K. D., Moffatt, S., Dave, M., & Pearce, M. S. | 2018 | Campbell, M. R., Mann, K. D., Moffatt, S., Dave, M., & Pearce, M. S. (2018). Social determinants of emotional well-being in new refugees in the UK. *Public Health*, *164*, 72-81. |  |  |
| Daou, K. R. A. Z. | 2022 | Daou, K. R. A. Z. (2022). Refugee Mothers Mental Health and Social Support Needs: A Systematic Review of Interventions for Refugee Mothers [Review]. *Europe's Journal of Psychology*, *18*(3), 337-349. |  |  |
| Disney, L. R., McPherson, J., & Jamal, Z. S. | 2021 | Disney, L. R., McPherson, J., & Jamal, Z. S. (2021). 'We know more than that': The Underemployment Experiences of College-educated Iraqi Refugees Living in the US. *Journal of Refugee Studies*, *34*(1), 1168-1184. |  |  |
| Dowling, A., Kunin, M., & Russell, G. | 2022 | Dowling, A., Kunin, M., & Russell, G. (2022). The impact of migration upon the perceived health of adult refugees resettling in Australia: a phenomenological study. *Journal of Ethnic and Migration Studies*, *48*(7), 1536-1553. |  |  |
| Greene, R. N. | 2019 | Greene, R. N. (2019). Kinship, friendship, and service provider social ties and how they influence well-being among newly resettled refugees. *Socius*, *5*. |  |  |
| Hawkes, C., Norris, K., Joyce, J., & Paton, D. | 2021 | Hawkes, C., Norris, K., Joyce, J., & Paton, D. (2021). Individuals of refugee background resettled in regional and rural Australia: A systematic review of mental health research. *The* *Australian Journal of Rural Health*, *29*(6), 850-864. |  |  |
| Jankovic-Rankovic, J., Oka, R. C., Meyer, J. S., Snodgrass, J. J., Eick, G. N., & Gettler, L. T. | 2022 | Jankovic-Rankovic, J., Oka, R. C., Meyer, J. S., Snodgrass, J. J., Eick, G. N., & Gettler, L. T. (2022). Transient refugees' social support, mental health, and physiological markers: Evidence from Serbian asylum centers. *American Journal of Human Biology*, *34*(7), e23747. |  |  |
| Kahil, R., Iqbal, M., & Maghbouleh, N. | 2022 | Kahil, R., Iqbal, M., & Maghbouleh, N. (2022). Grandmothers Behind the Scenes: Subordinate Integration, Care Work, and Power in Syrian Canadian Refugee Resettlement. *Refuge: Canada’s Journal on Refugees*, *38*(2), 1-18. |  |  |
| Kikhia, S., Gharib, G., Sauter, A., Vincens, N. C. L., & Loss, J. | 2021 | Kikhia, S., Gharib, G., Sauter, A., Vincens, N. C. L., & Loss, J. (2021). Exploring how Syrian women manage their health after migration to Germany: results of a qualitative study. *BMC Women’s Health*, *21*(1), 50. |  |  |
|  |  | (Continued) |  |  |
|  |  |  |  |  |
| Kindermann, D., Zeyher, V., Nagy, E., Brandenburg-Ceynowa, H., Junne, F., Friederich, H. C., Bozorgmehr, K., & Nikendei, C. | 2020 | Kindermann, D., Zeyher, V., Nagy, E., Brandenburg-Ceynowa, H., Junne, F., Friederich, H. C., Bozorgmehr, K., & Nikendei, C. (2020). Predictors of asylum seekers' mental health course in the early stages of resettlement: Results of a longitudinal study. *Journal of Psychosomatic Research*, *132*, 109977. |  |  |
| King, R. U., Heinonen, T., Uwabor, M., & Adeleye-Olusae, A. | 2017 | King, R. U., Heinonen, T., Uwabor, M., & Adeleye-Olusae, A. (2017). The Psychosocial Well-Being of African Refugees in Winnipeg: Critical Stressors and Coping Strategies. *Journal of Immigrant & Refugee Studies*, *15*(4), 345-365. |  |  |
| Mahadevan, R., & Jayasinghe, M. | 2024 | Mahadevan, R., & Jayasinghe, M. (2024). Are Factors Associated with Adult Refugees' Settlement different from Well-Being? A Longitudinal Study focusing on Gender and Age in Australia. *Journal of Social Policy*, 53(2), 470-490. |  |  |
| Martzoukou, K., & Burnett, S. | 2018 | Martzoukou, K., & Burnett, S. (2018). Exploring the everyday life information needs and the socio-cultural adaptation barriers of Syrian refugees in Scotland. *Journal of Documentation*, *74*(5), 1104-1132. |  |  |
| Miller, R., Tomita, Y., Ong, K. I. C., Shibanuma, A., & Jimba, M. | 2019 | Miller, R., Tomita, Y., Ong, K. I. C., Shibanuma, A., & Jimba, M. (2019). Mental well-being of international migrants to Japan: a systematic review. *BMJ Open*, *9*(11), e029988. |  |  |
| Modesti, C., & Talamo, A. | 2021 | Modesti, C., & Talamo, A. (2021). Defining Adjustment to Address the Missing Link between Refugees and Their Resettlement Communities. *International Journal of Environmental Research and Public Health*, *18*(18), Article 9902. |  |  |
| Mwanri, L., Miller, E., Walsh, M., Baak, M., & Ziersch, A. | 2023 | Mwanri, L., Miller, E., Walsh, M., Baak, M., & Ziersch, A. (2023). Social Capital and Rural Health for Refugee Communities in Australia. *International Journal of Environmental Research and Public Health*, *20*(3), 2378. |  |  |
| Nilsson, H., Saboonchi, F., Gustavsson, C., Malm, A., & Gottvall, M. | 2019 | Nilsson, H., Saboonchi, F., Gustavsson, C., Malm, A., & Gottvall, M. (2019). Trauma-afflicted refugees’ experiences of participating in physical activity and exercise treatment: a qualitative study based on focus group discussions. *European Journal of Psychotraumatology*, *10*(1), 1699327. |  |  |
| Njororai, F., & Lee, S. | 2018 | Njororai, F., & Lee, S. (2018). Social capital and health among Burundian refugees in the United States. *International Social Work*, *61*(6), 1116-1125. |  |  |
| Remennick, L. I. | 1999 | Remennick, L. I. (1999). Women of the "sandwich" generation and multiple roles: The case of Russian immigrants of the 1990s in Israel. *Sex Roles*, *40*, 347-378. |  |  |
| Remennick, L. I. | 2001 | Remennick, L. I. (2001). "All my life is one big nursing home": Russian immigrant women in Israel speak about double caregiver stress. *Women’s Studies International Forum*, *24*(6), 685-700. |  |  |
| Ryan, D., Tornberg-Belanger, S. N., Perez, G., Maurer, S., Price, C., Rao, D., Chan, K. C. G., & Ornelas, I. J. | 2021 | Ryan, D., Tornberg-Belanger, S. N., Perez, G., Maurer, S., Price, C., Rao, D., Chan, K. C. G., & Ornelas, I. J. (2021). Stress, social support and their relationship to depression and anxiety among Latina immigrant women. *Journal of Psychosomatic Research*, *149*, 110588. |  |  |
| Sá, F. H. de L., Waikamp, V., Freitas, L. H. M., & Baeza, F. L. C. | 2022 | Sá, F. H. de L., Waikamp, V., Freitas, L. H. M., & Baeza, F. L. C. (2022). Mental health outcomes in Syrian refugees: A systematic review. *International Journal of Social Psychiatry*, *68*(5), 933-953. |  |  |
| Shahzeidi, M., Stone, G., & Filiz, B. | 2023 | Shahzeidi, M., Stone, G., & Filiz, B. (2023). The Influence of Leisure on the Quality of Life of Refugees in Turkey. *Leisure Studies*, 43(2), 294-310. |  |  |
| Slade, N., & Borovnik, M. | 2018 | Slade, N., & Borovnik, M. (2018). 'Ageing out of place': Experiences of resettlement and belonging among older Bhutanese refugees in New Zealand. *New Zealand Geographer*, *74*(2), 101-108. |  |  |
| Sossou, M. A., Craig, C. D., Ogren, H., & Schnak, M. | 2008 | Sossou, M. A., Craig, C. D., Ogren, H., & Schnak, M. (2008). A Qualitative Study of Resilience Factors of Bosnian Refugee Women Resettled in the Southern United States. *Journal of Ethnic & Cultural Diversity in Social Work*, *17*(4), 365-385. |  |  |
|  |  | (Continued) |  |  |
|  |  |  |  | (Continued) |
| Sulaiman-Hill, C. M. R., & Thompson, S. C. | 2012 | Sulaiman-Hill, C. M. R., & Thompson, S. C. (2012). Afghan and Kurdish refugees, 8-20 years after resettlement, still experience psychological distress and challenges to wellbeing. *Australian and New Zealand Journal of Public Health*, *36*(2), 126-134. |  |  |
| Sulaiman-Hill, C. M. R., & Thompson, S. C. | 2012 | Sulaiman-Hill, C. M. R., & Thompson, S. C. (2012). "Thinking Too Much": Psychological distress, sources of stress and coping strategies of resettled Afghan and Kurdish refugees. *Journal of Muslim Mental Health*, *6*(2), 63-86. |  |  |
| Suto, M. J. | 2013 | Suto, M. J. (2013). Leisure Participation and Well-being of Immigrant Women in Canada. *Journal of Occupational Science*, *20*(1), 48-61. |  |  |
| Tinghög, P., Malm, A., Arwidson, C., Sigvardsdotter, E., Lundin, A., & Saboonchi, F. | 2017 | Tinghög, P., Malm, A., Arwidson, C., Sigvardsdotter, E., Lundin, A., & Saboonchi, F. (2017). Prevalence of mental ill health, traumas and postmigration stress among refugees from Syria resettled in Sweden after 2011: a population-based survey. *BMJ Open*, *7*(12), e018899. |  |  |
| Tip, L. K., Brown, R., Morrice, L., Collyer, M., & Easterbrook, M. J. | 2019 | Tip, L. K., Brown, R., Morrice, L., Collyer, M., & Easterbrook, M. J. (2019). Improving Refugee Well-Being With Better Language Skills and More Intergroup Contact. *Social Psychological and Personality Science*, *10*(2), 144-151. |  |  |
| Tippens, J. A., Roselius, K., Padasas, I., Khalaf, G., Kohel, K., Mollard, E., & Sheikh, I. | 2021 | Tippens, J. A., Roselius, K., Padasas, I., Khalaf, G., Kohel, K., Mollard, E., & Sheikh, I. (2021). Cultural Bereavement and Resilience in Refugee Resettlement: A Photovoice Study With Yazidi Women in the Midwest United States. *Qualitative Health Research*, *31*(8), 1486-1503. |  |  |
| Um, M. Y., Chi, I., Kim, H. J., Palinkas, L. A., & Kim, J. Y. | 2015 | Um, M. Y., Chi, I., Kim, H. J., Palinkas, L. A., & Kim, J. Y. (2015). Correlates of depressive symptoms among North Korean refugees adapting to South Korean society: The moderating role of perceived discrimination. *Social Science & Medicine*, *131*, 107-113. |  |  |
| Waardenburg, M., Visschers, M., Deelen, I., & van Liempt, I. | 2019 | Waardenburg, M., Visschers, M., Deelen, I., & van Liempt, I. (2019). Sport in liminal spaces: The meaning of sport activities for refugees living in a reception centre. *International Review for the Sociology of Sport*, *54*(8), 938-956. |  |  |
| Wachter, K., Dalpe, J., Bonz, A., Drozdowski, H., & Hermer, J. | 2021 | Wachter, K., Dalpe, J., Bonz, A., Drozdowski, H., & Hermer, J. (2021). A Scoping Review of Social Support Interventions with Refugees in Resettlement Contexts: Implications for Practice and Applied Research [Article]. *Journal of Immigrant &Refugee Studies*, *19*(4), 557-572. |  |  |
| Wachter, K., & Gulbas, L. E. | 2018 | Wachter, K., & Gulbas, L. E. (2018). Social support under siege: An analysis of forced migration among women from the Democratic Republic of Congo. *Social Science & Medicine*, *208*, 107-116. |  |  |
| Wanna, C. P., Seehuus, M., Mazzulla, E., & Fondacaro, K. | 2019 | Wanna, C. P., Seehuus, M., Mazzulla, E., & Fondacaro, K. (2019). A house is not a home: Modeling the effects of social support and connection within resettled refugee populations. *Journal of Community Psychol*, *47*(7), 1629-1644. |  |  |
| Yun, S., Ahmed, S. R., Hauson, A. O., & Al-Delaimy, W. K. | 2021 | Yun, S., Ahmed, S. R., Hauson, A. O., & Al-Delaimy, W. K. (2021). The Relationship Between Acculturative Stress and Postmigration Mental Health in Iraqi Refugee Women Resettled in San Diego, California. *Community Mental Health Journal*, *57*, 1111-1120. |  |  |
| Ziersch, A., Miller, E., Baak, M., & Mwanri, L. | 2020 | Ziersch, A., Miller, E., Baak, M., & Mwanri, L. (2020). Integration and social determinants of health and wellbeing for people from refugee backgrounds resettled in a rural town in South Australia: a qualitative study. *BMC Public Health*, *20*(1), 1700. |  |  |

**Table 3.** Social connectedness and outcome (well-being, life satisfaction, or resettlement) scales.

| **Author** | **Year** | **Social connectedness** | **Outcomes** |
| --- | --- | --- | --- |
| Abi Zeid Daou, K. R. | 2022 | Community building | Mental health/well-being |
| Al-Adhami, M., Berglund, E., Wångdahl, J., & Salari, R. | 2022 | Emotional support: “Do you have anybody whom you can share your deepest feelings with and confide in?“ Practical support: “How many people in your surroundings can you easily ask for help with everyday tasks” | General self-rated health (SRH): “How do you assess your overall health status? Psychological well-being: the General Health Questionnaire (GHQ-12) |
| Alexander, N., Mathilde, S., & Øivind, S. | 2021 | Social support: the ENRICHD Social Support Instrument (5 items) | Subjective Well-Being: WHO-5 well-being index (WHO-5) |
| Baeza-Rivera, M. J., Salazar-Fernández, C., Manríquez-Robles, D., Salinas-Oñate, N., & Smith-Castro, V. | 2022 | Perceived Social Support: Multidimensional Scale of Perceived Social Support (e.g., My colleagues try to help me; I can count on my colleagues when things go wrong; I can talk about my problems with my colleagues). | Mental Health Symptomatology: An ad hoc scale composed of three items was used to assess the frequency of depressive, anxiety, and stress symptoms (e.g., How often do you feel depressed, anxious, and/or stressed) |
| Bedaso, A., & Duko, B. | 2022 | Social Support (Emotional support) | Depression |
| Brailovskaia, J., Schönfeld, P., Kochetkov, Y., & Margraf, J. | 2019 | Social support: F-SozU K-14 | The Depression Anxiety Stress Scales 21 (DASS-21; Lovibond and Lovibond 1995), a self-report questionnaire consisting of three 7-item subscales, measured depression, anxiety, and stress symptoms (negative metal health) |
| Campbell, M. R., Mann, K. D., Moffatt, S., Dave, M., & Pearce, M. S. | 2018 | Proxy for networks: frequency of contact with relatives, friends, their own national or ethnic group and place of worship in the UK | Emotional well-being: The question used was ‘During the past 4 weeks, how much have you been bothered by emotional problems (such as feeling worried, stressed or depressed)? from the Short Form-36 (SF-36) Health Survey Questionnaire |
| Disney, L. R., McPherson, J., & Jamal, Z. S. | 2021 | Formal and informal network (e.g., refugee community networks, resettlement agency) | Resettlement: "Has your employment experience in the US positively/negatively affected your resettlement in the US? In what ways?" Life satisfaction: "Has your employment experience in the US positively/negatively affected your life satisfaction? In what ways?" |
| Dowling, A., Kunin, M., & Russell, G. | 2022 | Social supports (family, government, broad) | Health: How was my health as a result of my pre-/peri/post-migration experiences? |
|  |  |  | (Continued) |
| Greene, R. N. | 2019 | Social ties (e.g., Kinship ties, Friendship ties, Social service provider ties). (1) “Are there people (adults) in your community with whom you discuss important matters?” (2) “Are there people (adults) in your community who you have asked for advice or help in getting things done in the United States?” and (3) Are there people (adults) in your community whom you ask for advice or help when you are not feeling good about yourself or your situation?” | Emotional distress: the Hopkins Symptoms Checklist–25 (HSCL-25, without loss of sexual interest or pleasure item), Psychological Quality of life: a modified version of the World Health Organization Quality of Life assessment (WHOQOL) |
| Hawkes, C., Norris, K., Joyce, J., & Paton, D. | 2021 | Connection with individuals from same culture of origin,  Host community support | Mental health |
| Jankovic-Rankovic, J., Oka, R. C., Meyer, J. S., Snodgrass, J. J., Eick, G. N., & Gettler, L. T. | 2022 | Social supports: Friendship scale (e.g., Do you have people you can trust with your intimate thoughts and feelings?, Do you have people you can ask for advice from when you have a problem?) | Subjective distress by traumatic events: 22-item Impact of Event Scale-Revised (IESR). Trauma-related mental and physical health: Refugee Helath Screener 15 (RHS-15). Psycho-social stress: 10-item Cohen Perceived Stress Scale (PSS). |
| Kahil, R., Iqbal, M., & Maghbouleh, N. | 2022 | Informal and formal supports from co-ethic community networks and government resettlement services | resettlement |
| Kikhia, S., Gharib, G., Sauter, A., Vincens, N. C. L., & Loss, J. | 2021 | Social support | Health: “How do Syrian immigrant women experience managing their own and their family lies’ health, after their settlement in private accommodation in Germany?” |
| Kindermann, D., Zeyher, V., Nagy, E., Brandenburg-Ceynowa, H., Junne, F., Friederich, H. C., Bozorgmehr, K., & Nikendei, C. | 2020 | Sense of Coherence: the Leipzig Short Scale of the Sense of Coherence Scale (SOC-9 L) | Posttraumatic stress disorder: Primary Care PTSD Screening (PC-PTSD-5), Depressive disorders: Patient Health Questionnaire (PHQ-2), General anxiety disorders: General Anxiety Disorder Questionnaire (GAD-2), Panic disorders: Patient Health Questionnaire for Panic Disorder (PHQ-PD), Mental well-being: the World Health Organization's Well-Being Index (WHO-5), Emotion Regulation: Emotion Regulation Questionnaire (ERQ-10) |
| King, R. U., Heinonen, T., Uwabor, M., & Adeleye-Olusae, A. | 2017 | Social network, social support | mental health |
|  |  |  | (Continued) |
| Mahadevan, R., & Jayasinghe, M. | 2024 | Received ethnic support, Sense of belonging | Settlement Experience: Overall, your experience of settling in Australia so far has been Life satisfaction: Thinking about your own life and personal circumstances, how satisfied are you with your life as a whole?’ |
| Martzoukou, K., & Burnett, S. | 2018 | Social support, engagement in community | Resettlement |
| Miller, R., Tomita, Y., Ong, K. I. C., Shibanuma, A., & Jimba, M. | 2019 | Social networks, social support | Mental well-being |
| Modesti, C., & Talamo, A. | 2021 | Sense of community, Co-ethic community, resettlement community | Well-being, Resettlement |
| Mwanri, L., Miller, E., Walsh, M., Baak, M., & Ziersch, A. | 2023 | Social ties (Bonding ties & Binding ties), Social connection | Social determinants of health (SDH): a framework to consider the social, economic and cultural factors that impact health |
| Nilsson, H., Saboonchi, F., Gustavsson, C., Malm, A., & Gottvall, M. | 2019 | Physical activity (Adjunctive group-based treatment e.g. warm-water pool training, aerobics classes, yoga, gym training, ball sport activities, Basic Body Awareness Training) | Physical and mental health (e.g., Initial barriers of participation in the treatment, the process of overcoming such barriers, and experiences of reduced symptoms and increased wellbeing) |
| Njororai, F., & Lee, S. | 2018 | Social capital: Bonding capital A higher score indicates more contact with network individuals within the same ethnic community. Bridging capital, A higher score indicates more contact with network individuals from different ethnic groups in the wider community | Perceived health status: Very good/good = 1, fair/poor = 0. Physical health conditions: In numbers. |
| Remennick, L. I. | 1999 | Co-ethnic community network | Well-being |
| Remennick, L. I. | 2001 | Co-ethnic community network | Health |
| Ryan, D., Tornberg-Belanger, S. N., Perez, G., Maurer, S., Price, C., Rao, D., Chan, K. C. G., & Ornelas, I. J. | 2021 | Social ties: The Lubben Social Network Scale Participants reported the number and frequency of contact they had with friends, family, and relatives, and the degree to which they feel supported by these contacts in this 6-item scale. Social support: Annreviated version of the Medical Outcome Study Social Support Measure. This instrument is comprised of three subscales: Emotional Support, Affectionate Support, Positive Social interaction | Depressive symptoms: the Patient Health Questionnaire 9 (PHQ-9). Anxiety symptoms: the 7-item Generalized. Anxiety Disorders-7 (GAD-7) scale. Perceived stress scale (PSS): 4-item (e.g., how often they experienced certain overwhelming thoughts or feelings) |
| Sá, F. H. de L., Waikamp, V., Freitas, L. H. M., & Baeza, F. L. C. | 2022 | Social support (peers) | Mental health (psychiatric disorders,  mainly PTSD, anxiety, and depression), well-being |
|  |  |  | (Continued) |
| Shahzeidi, M., Stone, G., & Filiz, B. | 2023 | Leisure availability was measured using 5-pt Likert items addressing access (e.g. ‘Do you have access to . . .?’) and ability (e.g. ‘Is it possible for you to . . .?’). Leisure participation was measured using 5-pt Likert items addressing frequency (e.g. ‘How often do you participate in. . .)?’ and time (i.e. ‘How much time do you spend on . . .?’). | The questionnaire reflected QOL measurement items developed and validated by the World Health Organization (WHOQOL-BREF) |
| Slade, N., & Borovnik, M. | 2018 | Social bonds (Family, community, and cultural) | Resettlement |
| Sossou, M. A., Craig, C. D., Ogren, H., & Schnak, M. | 2008 | Community social support | Resettlement |
| Sulaiman-Hill, C. M. R., & Thompson, S. C. | 2012 | Social support (from family), social contact | Psychological well-being: Kessler-10 Psychological Distress Scale (K-10) |
| Sulaiman-Hill, C. M. R., & Thompson, S. C. | 2012 | family support (Loss of traditional support base, family separation) | Quality of life |
| Suto, M. J. | 2013 | Participation in leisure | Health |
| Tinghög, P., Malm, A., Arwidson, C., Sigvardsdotter, E., Lundin, A., & Saboonchi, F. | 2017 | social support (as a proxy): Often felt sad because not reunited with family member | Anxiety & Depression: HSCL-25, PTSD: HTQ Subjective well-being: WHO-5 |
| Tip, L. K., Brown, R., Morrice, L., Collyer, M., & Easterbrook, M. J. | 2019 | Contact with the British majority: How often do you interact with British people?” and “In the last month, how many times have you interacted with British people?” | Well-being: Thinking about yourself and how you normally feel, to what extent do you generally feel. ... alert/inspired/determined/attentive/active |
| Tippens, J. A., Roselius, K., Padasas, I., Khalaf, G., Kohel, K., Mollard, E., & Sheikh, I. | 2021 | Ethnic community support | Health and well-being (e.g., “What does health and well-being mean to you and your community?” and “What are your health priorities?”) |
| Um, M. Y., Chi, I., Kim, H. J., Palinkas, L. A., & Kim, J. Y. | 2015 | The Sociocultural Adaptation: NK refugees' social adaptation, cultural adaptation, interpersonal adaptation, and sense of belongingness (e.g., “I cannot feel a sense of belonging in South Korean society.”) | Depressive symptomatology: the 20-item Center for Epidemiologic Studies Depression Scale (CES-D) |
| Waardenburg, M., Visschers, M., Deelen, I., & van Liempt, I. | 2019 | sport activities (physical) | Well-being |
| Wachter, K., Dalpe, J., Bonz, A., Drozdowski, H., & Hermer, J. | 2021 | social support | Resettlement |
| Wachter, K., & Gulbas, L. E. | 2018 | social networks | Well-being and health |
|  |  |  | (Continued) |
| Wanna, C. P., Seehuus, M., Mazzulla, E., & Fondacaro, K. | 2019 | community support: "Satisfaction with community support" | Traumatic stress: Harvard Trauma Questionnaire (HTQ), Anxiety & Depressive symptoms: Hopkins Symptom Checklist (HSCL) |
| Yun, S., Ahmed, S. R., Hauson, A. O., & Al-Delaimy, W. K. | 2021 | Acculturative Stress: The Social, Attitudinal, Familial, and Environmental Acculturative Stress scale (SAFE). (e.g., I don't feel at home, I don't have any close friends, Loosening the ties with my country is difficult.) | Anxiety (first 10) & Depression (last 15 items): The Hopkins Symptoms Checklist (HSCL-25) |
| Ziersch, A., Miller, E., Baak, M., & Mwanri, L. | 2020 | Safety and belonging, social networks and support | Social Determinants of Health (SDH), Well-being |

**Table 4.** Main findings

| **Author** | **Year** | **Main Findings (Direct)** | **Main Findings (Indirect)** |  |
| --- | --- | --- | --- | --- |
| Abi Zeid Daou, K. R. | 2022 | Community building and safe community spaces to connect with fellow refugees and simply talk, laugh, and bond which also provided them a space for emotional support and information access, and a network are protective factors for refugee mothers their post resettlement needs and improving their mental health, well-being. |  |  |
| Al-Adhami, M., Berglund, E., Wångdahl, J., & Salari, R. | 2022 | Lack of emotional social support was associated with poor self-rated health (SRH). Lack of emotional social support was associated with poor psychological well-being. Lack of practical social support was associated with poor self-rated health (SRH). |  |  |
| Alexander, N., Mathilde, S., & Øivind, S. | 2021 |  | Social support appeared to buffer the adverse effects of financial strain on SWB. |  |
| Baeza-Rivera, M. J., Salazar-Fernández, C., Manríquez-Robles, D., Salinas-Oñate, N., & Smith-Castro, V. | 2022 | There was no direct effect of perceived social support on mental health. | Perceived social support was associated with acculturative stress by reducing mental health symptomatology. |  |
| Bedaso, A., & Duko, B. | 2022 | The rates of depression were significantly lower among migrants or refugees with satisfactory emotional support or high social support. A refugee or migrant with lower emotional support had a higher odd of depression. |  |  |
| Brailovskaia, J., Schönfeld, P., Kochetkov, Y., & Margraf, J. | 2019 | Depression symptoms were significantly negatively associated with social support among Russian migrants (p < .01). Depression symptoms were marginally negatively associated with social support among USA migrants (p < .10). |  |  |
|  |  |  | (Continued) |  |
| Campbell, M. R., Mann, K. D., Moffatt, S., Dave, M., & Pearce, M. S. | 2018 | Refugees who had infrequent contact with relatives (as proxy of community networks) had worse emotional well-being. |  |  |
| Disney, L. R., McPherson, J., & Jamal, Z. S. | 2021 |  | Counsel from informal community networks regarding employment was associated with assistance in finding jobs, leading to increased life satisfaction and a positive resettlement experience. Counsel from formal networks such as resettlement agencies had a limited impact on life satisfaction and resettlement experiences. |  |
| Dowling, A., Kunin, M., & Russell, G. | 2022 | Pre-migration: Family support has a positive impact on health. Peri-migration (transit): Broad social support: Social support networks included family, friends and community groups, which provided emotional and material support alleviated distress. Post-migration: Australian government were viewed positively by participants and were linked to the alleviation of health issues. | Participants described links between support from broad social networks and positive health and well-being. |  |
| Greene, R. N. | 2019 | Family ties were not related to emotional distress. Friendship ties were not related to emotional distress. Social service provider ties were more likely to have greater emotional distress. Family ties are associated with greater psychological quality of life |  |  |
| Hawkes, C., Norris, K., Joyce, J., & Paton, D. | 2021 | Disconnection with family had on the mental health of individuals of a refugee background resettled in a rural or regional location. Support from the host community decreased stress. |  |  |
|  |  |  | (Continued) |  |
| Jankovic-Rankovic, J., Oka, R. C., Meyer, J. S., Snodgrass, J. J., Eick, G. N., & Gettler, L. T. | 2022 | In OLS regression models of refugees' mental and physical health (RHS-15 scores), post-traumatic stress-related symptomology (IES-R scores), and recent perceived stress (PSS negative sub-scale), refugees with lower social support reported poorer mental well-being. |  |  |
| Kahil, R., Iqbal, M., & Maghbouleh, N. | 2022 | The respondents received both informal and formal supports from co-ethnic community social networks and government resettlement services that help the resettlement process. |  |  |
| Kikhia, S., Gharib, G., Sauter, A., Vincens, N. C. L., & Loss, J. | 2021 | Lack of social support had a negative impact on chronic stress. |  |  |
| Kindermann, D., Zeyher, V., Nagy, E., Brandenburg-Ceynowa, H., Junne, F., Friederich, H. C., Bozorgmehr, K., & Nikendei, C. | 2020 | Sense of Coherence (SOC) was found to be no predictor of mental health |  |  |
| King, R. U., Heinonen, T., Uwabor, M., & Adeleye-Olusae, A. | 2017 | Erosion of social and familial networks and supports identified as a risk factors for mental health. |  |  |
| Mahadevan, R., & Jayasinghe, M. | 2024 | A sense of belonging was positively associated with settlement experience (SE) in all cohorts. Ethnic support had a positive impact on SE only older cohort (Age =>38). A sense of belonging was positively associated with Life satisfaction (LS) in all cohorts. Ethnic support is negatively associated with Life satisfaction (Ls) in full and male cohort. |  |  |
| Martzoukou, K., & Burnett, S. | 2018 | Organising social activities and helping Syrian families build social and emotional bonds with other local Scottish families was very important for well-being and resettlement process. | Learning the English language was a significant priority for the Syrian New Scots because it was directly related to their potential for community integration, helping to reduce feelings of isolation and providing more opportunities to engage in the community. |  |
|  |  |  | (Continued) |  |
| Miller, R., Tomita, Y., Ong, K. I. C., Shibanuma, A., & Jimba, M. | 2019 | Lack of social support were barrier to mental well-being. Social support and networks were facilitators of mental well-being |  |  |
| Modesti, C., & Talamo, A. | 2021 | Sense of community may increase refugees’ psychological well-being. Co-ethnic networks foster life satisfaction. Resettlement community foster resettlement. |  |  |
| Mwanri, L., Miller, E., Walsh, M., Baak, M., & Ziersch, A. | 2023 | Communities were able to access resources associated with a sense of belonging and welcome and to access information about housing, employment, and health care mainly from bonding ties within their own cultural community. There was some evidence of resources gained through bridging ties with members of the broader community through church and school settings; however, strengthening social connection between refugee and migrant arrivals and the majority population is likely to be beneficial for all. |  |  |
| Nilsson, H., Saboonchi, F., Gustavsson, C., Malm, A., & Gottvall, M. | 2019 | Physical activity reduced symptoms of physical and mental distress. Physical activity increased well-being |  |  |
| Njororai, F., & Lee, S. | 2018 | Quantitative findings do not show a significant relationship between social capital (both bonding and bridging) and health (both perceived health status and number of health conditions). Qualitative findings: Lack of social capital affected their health status (including being depressed and lonely, and caused them more stress). |  |  |
| Remennick, L. I. | 1999 | Informal and formal Russian(co-ethic) networks are very helpful for the elders, discharging of negative feelings. Israeli friends became an important source of guidance and practical aid in the matters (resettlement). |  |  |
|  |  |  | (Continued) |  |
| Remennick, L. I. | 2001 | Informal and formal Russian(co-ethic) networks are very helpful for the elders, discharging of negative feelings. Israeli friends became an important source of guidance and practical aid in the matters (resettlement). Informal co-ethic social network mentioned in the interviews as key sources of information, emotional support and instrumental help in case of need. |  |  |
| Ryan, D., Tornberg-Belanger, S. N., Perez, G., Maurer, S., Price, C., Rao, D., Chan, K. C. G., & Ornelas, I. J. | 2021 | Social support received through positive social interactions was associated with decreased anxiety symptoms, while social ties/ Emotional/ Affectionate support was not significant. Social support (all sub-category: social ties/ Emotional/ Affectionate support/ Positive social interaction) was not significantly associated with depression |  |  |
| Sá, F. H. de L., Waikamp, V., Freitas, L. H. M., & Baeza, F. L. C. | 2022 | Lack of social support had a positive association with a major depressive disorder. Lack of social support had a positive association with PTSD. Social support among peers and social integration with the host country community provide global well-being |  |  |
| Shahzeidi, M., Stone, G., & Filiz, B. | 2023 | Leisure participation showed a strong association with Quality of life. |  |  |
| Slade, N., & Borovnik, M. | 2018 | Family, Community, and Cultural bonds had a positive impact on elders' resettlement process. |  |  |
| Sossou, M. A., Craig, C. D., Ogren, H., & Schnak, M. | 2008 | Community social support had a positive impact on successful resettlement |  |  |
| Sulaiman-Hill, C. M. R., & Thompson, S. C. | 2012 | Family separation and lack of support increased stress. An examination of the coping strategies employed  by females suggests the importance of social contact, especially with other women. |  |  |
|  |  |  | (Continued) |  |
| Sulaiman-Hill, C. M. R., & Thompson, S. C. | 2012 | Loss of family support (Lack of extended family) had a negative impact on Quality of life. |  |  |
| Suto, M. J. | 2013 | Many women defined leisure broadly as whatever supported their physical and emotional health. |  |  |
| Tinghög, P., Malm, A., Arwidson, C., Sigvardsdotter, E., Lundin, A., & Saboonchi, F. | 2017 | Often felt sad because not reunited with family members generally exhibited the weakest association with mental ill health which is depression, low SWB, PTSD (However, certain sensitivity analyses, especially those with a lenient threshold, did not substantiate this association). Often sad due to not being reunited with family members, which was not significantly associated with anxiety |  |  |
| Tip, L. K., Brown, R., Morrice, L., Collyer, M., & Easterbrook, M. J. | 2019 | Contact with British people was positively associated with well-being |  |  |
| Tippens, J. A., Roselius, K., Padasas, I., Khalaf, G., Kohel, K., Mollard, E., & Sheikh, I. | 2021 | Older women relied on others from their ethnic community support to enhance the well-being. Separation from family members and loved ones was a central cause of distress among participants. |  |  |
| Um, M. Y., Chi, I., Kim, H. J., Palinkas, L. A., & Kim, J. Y. | 2015 | Poor sociocultural adaptation was associated with more depressive symptoms. | Perceived discrimination moderated the association between sociocultural adaptation and depressive symptoms among NK refugees. |  |
| Waardenburg, M., Visschers, M., Deelen, I., & van Liempt, I. | 2019 | Sport is one such an activity - people in the reception centre practice sport so they can relax; it functions as a form of relief for them. |  |  |
| Wachter, K., Dalpe, J., Bonz, A., Drozdowski, H., & Hermer, J. | 2021 | Social support intervention that facilitates various forms of social support had a positive impact on resettlement process. Notable programming approaches included group formats, structured/unstructured programming, shared spaces, peer support, technology, and family as a source of support. |  |  |
|  |  |  | (Continued) |  |
| Wachter, K., & Gulbas, L. E. | 2018 | Well-being is impacted by the loss of resources embedded in relational networks during post-resettlement phase. Women struggle with being separated from their family, their family unit being torn apart, and learning to stand alone and rely on themselves. They benefit from being at work and feeling part of a group or community. |  |  |
| Wanna, C. P., Seehuus, M., Mazzulla, E., & Fondacaro, K. | 2019 | The satisfaction with community support was not significantly related to depressive symptoms. | English language fluency is a moderator those who were fluent in English and satisfied with community support reported significantly fewer depressive symptoms. |  |
| Yun, S., Ahmed, S. R., Hauson, A. O., & Al-Delaimy, W. K. | 2021 | An increase in acculturative stress increased the likelihood for depression and anxiety. |  |  |
| Ziersch, A., Miller, E., Baak, M., & Mwanri, L. | 2020 | Social connectedness through both within their cultural or ethnic communities and through settlement services, was a key element for positive integration and health outcomes. Spiritual support and friendship, along with practical help from various social connections, helped people find services or resources, or navigate other settlement issues. |  |  |
|  |  |  |  |  |

**Figure 1.** Year of publication (Cumulative)


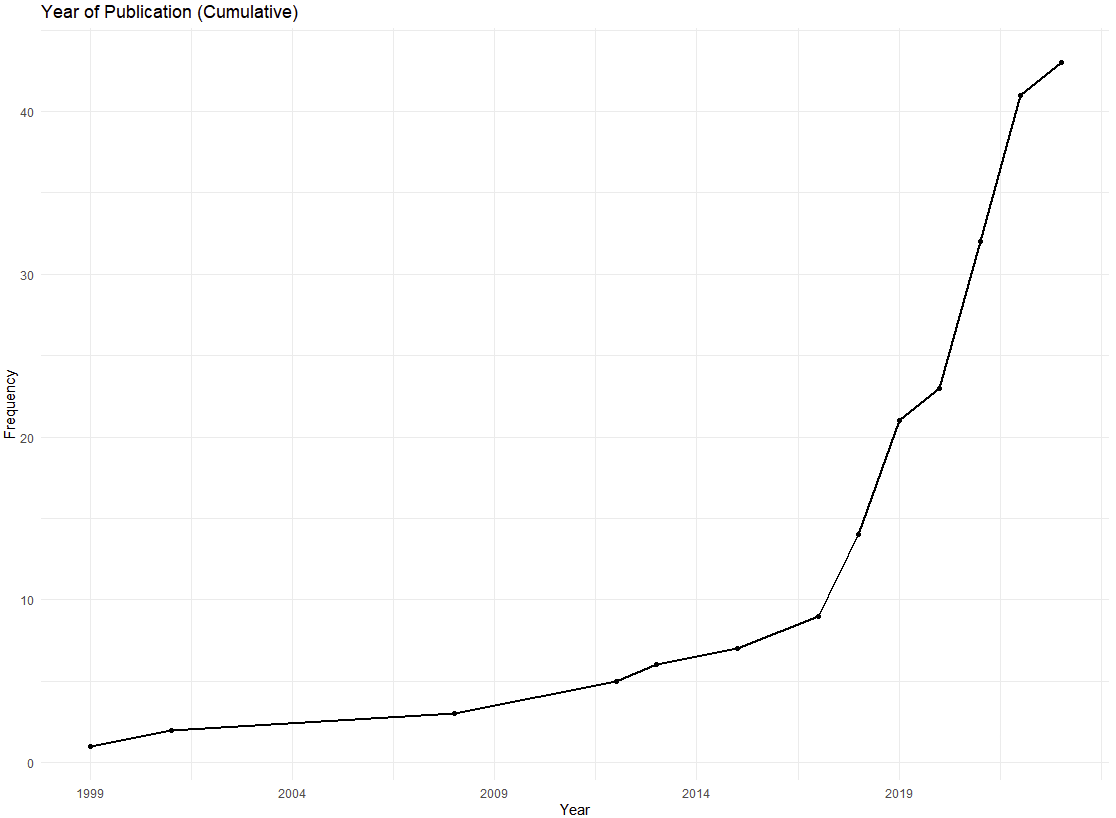

Supplement: Supplementary file 1 — Supplementary file1 (DOCX 90 KB) [file 40615_2024_2036_MOESM1_ESM.docx]
